# A regional study of the genus *Phyllopsora* (Ramalinaceae) in Asia and Melanesia

**DOI:** 10.3897/mycokeys.53.33425

**Published:** 2019-05-29

**Authors:** Sonja Kistenich, Mika Bendiksby, Charles S. Vairappan, Gothamie Weerakoon, Siril Wijesundara, Patricia A. Wolseley, Einar Timdal

**Affiliations:** 1 Natural History Museum, University of Oslo, 0318 Oslo, Norway University of Oslo Oslo Norway; 2 NTNU University Museum, Norwegian University of Science and Technology, 7012 Trondheim, Norway Norwegian University of Science and Technology Trondheim Norway; 3 Institute for Tropical Biology and Conservation, Universiti Malaysia Sabah, 88400 Kota Kinabalu, Sabah, Malaysia Universiti Malaysia Sabah Kota Kinabalu Malaysia; 4 Department of Life Sciences, The Natural History Museum, London SW75BD, UK The Natural History Museum London United Kingdom; 5 National Institute of Fundamental Studies, 20000 Kandy, Sri Lanka National Institute of Fundamental Studies Peradeniya Sri Lanka

**Keywords:** Malaysia, Sri Lanka, Thailand, rainforest, TLC, phylogeny, identification key

## Abstract

*Phyllopsora* is a crustose to squamulose lichen genus inhabiting the bark of trees in moist tropical forests and rainforests. Species identification is generally challenging and is mainly based on ascospore morphology, thallus morphology and anatomy, vegetative dispersal units, and on secondary chemistry. While regional treatments of the genus have been conducted for Africa, South America and Australia, there exists no study focusing on the Asian and Melanesian species. Previously, 24 species of *Phyllopsora* s. str. have been reported from major national studies and checklists representing 13 countries. We have studied herbarium material of 625 *Phyllopsora* specimens from 18 countries using morphology, anatomy, secondary chemistry, and molecular data to investigate the diversity of *Phyllopsora* species in Asia and Melanesia. We report the occurrence of 28 species of *Phyllopsora* including the following three species described as new to science: *P.sabahana* from Malaysia, *P.siamensis* from Thailand and *P.pseudocorallina* from Asia and Africa. Eight species are reported as new to Asia. A key to the Asian and Melanesian species of *Phyllopsora* is provided.

## Introduction

The genus *Phyllopsora* Müll. Arg. consists of 54 crustose or squamulose species (Kistenich et al. in press). They grow mostly on bark of trees in (sub-)tropical rainforests or moist woodlands. The genus was described in 1894 from New Zealand (Müller 1894), but the first modern revision of the pantropical genus was conducted 87 years later by Swinscow and Krog (1981) focusing on the East African species. Ten years later, Brako (1991) monographed the Neotropical species, while Elix (2009) summarized the Australian species and their occurrence. Additional reports and regional studies of the genus and its species, distribution can be found from Eastern Africa (Timdal and Krog 2001), Peru (Timdal 2008b) and the West Indies (Timdal 2011). From Asia, however, only a few reports exist for selected countries. Upreti et al. (2002) listed five *Phyllopsora* species from India. Later, Mishra et al. (2011) described two species and one variety from India as new to science. Recently, Kondratyuk et al. (2016) described a new species from South Korea. Phyllopsoroid specimens have been reported in additional checklists and geographical studies from, for example, Bangladesh (Aptroot and Iqbal 2011), Northeast India (Logesh et al. 2017), Sri Lanka (Weerakoon and Aptroot 2014), South Korea (Joshi et al. 2011) and Thailand (Aptroot et al. 2007). A general Asian, transnational study focusing on *Phyllopsora* has to date not been published. So far, 24 of the 54 accepted *Phyllopsora* species have been reported to occur in Asia and Melanesia (Table 1). An additional nine species reported from Asia represent either synonyms or have recently been excluded from the genus (Kistenich et al. in press; 2018b; Table 1).

Species of *Phyllopsora* are generally challenging to identify by morphology only. In a molecular phylogeny of the lichen family Ramalinaceae C. Agardh, Kistenich et al. (2018b) showed the genus *Phyllopsora* to be polyphyletic. Consequently, they excluded ten species from the genus. Three of the excluded species most likely belong in the family Malmideaceae Kalb, Rivas Plata & Lumbsch. An additional three of the excluded species were transferred to the new genus *Parallopsora* Kistenich, Timdal & Bendiksby. The species of *Parallopsora* grouped together in a poorly resolved clade with a number of tropical genera, such as *Eschatogonia* Trevis., *Krogia* Timdal and *Physcidia* Tuck. (Kistenich et al. 2018b). Little is known about these genera in Asia, which are generally very similar to *Phyllopsora* in their macromorphology. They often differ, however, from *Phyllopsora* in ascospore size and arrangement, presence of prothallus, thallus construction and chemistry (Kalb and Elix 1995; Kistenich et al. 2018b; Timdal 2008a; 2009). Recently, Kistenich et al. (2018a) described three new species of *Krogia* from Asia and Oceania, which were all tentatively identified as *Phyllopsora* sp., indicating the morphological similarity between these two genera.

The scope of the present study is to revise Asian and Melanesian *Phyllopsora* specimens mainly collected between 1990 and 2017 by the authors. Herein, we provide an overview of the species of *Phyllopsora* occurring in the Asian countries with an updated taxonomy based on multiple sources of evidence, including DNA sequence data. We describe three new species and provide a key to the Asian and Melanesian species of *Phyllopsora*.

**Table 1.** Species of *Phyllopsora* reported from Asia and Melanesia.

Species	Authorship	Cambodia	China	Fiji	India	Indonesia	Japan	Malaysia	Nepal	New Caledonia	Papua New Guinea	Philippines	Solomon	South Korea	Sri Lanka	Taiwan	Thailand	Vanuatu	Vietnam
**Accepted species**																			
* P. africana *	Timdal & Krog					**	**	**			*	**	**		**		**	*	
* P. breviuscula *	(Nyl.) Müll. Arg.				* [10]					**		**			** [17]				*
* P. buettneri *	(Müll. Arg.) Zahlbr.		*		[16]		**				* [1, 5, 15]	[5]			*	[3]	** [6]		[4]
* P. castaneocincta *	(Hue) Kistenich & Timdal	*			* [16]		* T	**	**	*	*	* T	*		** [17]	*	**		
* P. chodatinica *	Elix							**		**	* [5]							*	
* P. cinchonarum *	(Fée) Timdal						** [12]									* T	** [6]		
* P. confusa *	Swinscow & Krog				[10]	*	*	**			** [1, 15]				** [17]	*	**		
* P. cuyabensis *	(Malme) Zahlbr.																**		
* P. dolichospora *	Timdal & Krog						*				**				** [17]				
* P. foliata *	(Stirt.) Zahlbr.						**								** [17]				
* P. furfuracea *	(Pers.) Zahlbr.				[10]	[5]				[8]	* [2, 7]	T			* [17]	[3]	** [6, 19]		[4]
* P. gossypina *	(Sw.) Kistenich et al.						[12]	[13]		[8]	[14, 15]				** [17]		[6]		[4]
* P. halei *	(Tuck.) Zahlbr.										[1, 2]				**	[3]			
* P. himalayensis *	G.K. Mishra et al.				T														
* P. isidiosa *	Kistenich & Timdal					**		**	**			**					**		
* P. kalbii *	Brako				[10]												**		
* P. loekoesii *	S.Y. Kondr. et al.						**		**					* T					
* P. longiuscula *	(Nyl.) Zahlbr.														**		**		*
* P. mediocris *	Swinscow & Krog														*				
* P. neofoliata *	Elix														*				
* P. parvifolia *	(Pers.) Müll. Arg.		[18]		[16]					[7, 8]	[14]				*				
* P. parvifoliella *	(Nyl.) Müll. Arg.					**					*	*					**		
* P. porphyromelaena *	(Vain.) Zahlbr.			*	* [10]	*	**	**		*	*	* T		*	**	* T	**		
* P. pseudocorallina *	Kistenich & Timdal	**						**			**								
* P. sabahana *	Kistenich & Timdal							**											
* P. santensis *	(Tuck.) Swinscow & Krog						[12]				[2]	[5]					*		
* P. siamensis *	Kistenich & Timdal																**		
* P. subhispidula *	(Nyl.) Kalb & Elix														**				
**Reported, not confirmed species**																			
* P. chlorophaea *	(Müll. Arg.) Zahlbr.				[10]											[3]			
* P. corallina *	(Eschw.) Müll. Arg.				[9, 16]									[11]	[17]	[3]			[4]
* P. isidiotyla *	(Vain.) Riddle				[10]														
* P. mauritiana *	(Taylor) Swinscow & Krog				[10]														
* P. nemoralis *	Timdal & Krog				[10]														
* P. pyxinoides *	(Nyl.) Kistenich et al.																[6, 19]		
* P. swinscowii *	Timdal & Krog				[10]														
**Excluded species**																			
* P. catervisorediata *	G.K. Mishra et al.				T														
* P. densiflorae *	(Vain.) Gotth. Schneid.						T												
* P. griseocastanea *	(Vain.) Gotth. Schneid.											T							
* P. manipurensis *	(Müll. Arg.) Müll. Arg.				T														
* P. subcrustacea *	(Malme) Brako				[10]														
* P. viridis *	Paulson																T		
* P. borbonica *	Timdal & Krog														[17]				
* P. sorediata *	(Aptroot & Sparrius) Timdal																[6]		
* P. soralifera *	Timdal				[9]														

T: Type material (of the accepted name or a synonym); *: Identified in this study, based on morphology/chemistry; **: Identified in this study, based on DNA; [1]: Aptroot 1997; [2]: Aptroot et al. 1997; [3]: Aptroot and Sparrius 2003; [4]: Aptroot and Sparrius 2006; [5]: Brako 1991; [6]: Buaruang et al. 2017; [7]: Elix 2009; [8]: Elix and McCarthy 1998; [9]: Logesh et al. 2017; [10]: Mishra et al. 2011; [11]: Moon 2013; [12]: Ohmura and Kasiwadani 2018; [13]: Sipman 1993; [14]: Streimann 1986; [15]: Streimann and Sipman 1994; [16]: Upreti et al. 2002; [17]: Weerakoon and Aptroot 2014; [18]: Wei 1991; [19]: Wolseley et al. 2002.

## Materials and methods

### The specimens

We investigated material from 18 different countries in Asia and Melanesia (Table 1) based on herbarium collections made mainly between 1990 and 2017. Older material of *Phyllopsora* is generally not suitable for DNA sequencing (Kistenich et al. in press). In addition to material from our own herbaria directly available to us (BM, BORH, O, PDA), we received loans from the institutional herbaria B, E, H, TNS, and UPS, as well as from the private herbarium of P. Diederich. In total, we investigated 908 specimens of *Phyllopsora* and related genera. Author names for the studied species are provided in Tables 1 and 2.

The definition of Melanesia follows the United Nations geoscheme for Oceania as devised by the United Nations Statistics Division based on the M49 coding classification (https://unstats.un.org/unsd/methodology/m49/). Accordingly, it includes the five countries Fiji, New Caledonia, Papua New Guinea, Solomon Islands, and Vanuatu.

### Morphology and secondary chemistry

All specimens were studied morphologically and when necessary, also anatomically. Microscope sections were prepared using a freezing microtome and mounted in water, 10% KOH (K), lactophenol cotton blue, and a modified Lugol’s solution in which water was replaced by 50% lactic acid. The types of upper cortex referred to in this paper (types 1 and 2) are those described by Swinscow and Krog (1981). Amyloid reactions in the apothecium were observed in the modified Lugol’s solution after pretreatment in K, and crystals of lichen substances were observed using polarized light. Ascospore measurements are given as X ± 1.5 × SD rounded to 0.5 µm, where X is the arithmetic mean and SD the standard deviation.

We performed thin-layer chromatography (TLC) as routine investigation for identification of lichen substances in accordance with the methods of Culberson (1972), modified by Menlove (1974) and Culberson and Johnson (1982). Generally, we examined the acetone-extracts in solvent system B’; fatty acids were not examined. In difficult cases, we additionally used solvent systems A and C for lichen substance identification.

### Molecular methods and phylogenetic analysis

For DNA extraction, PCR amplification and DNA sequencing of the mitochondrial ribosomal small subunit (mtSSU) and the nuclear ribosomal internal transcribed spacer region (ITS: ITS1, 5.8S, ITS2), we followed the protocols outlined in Kistenich et al. (2018a). For sequence assembly and preliminary alignment, we used Geneious R9 (Kearse et al. 2012).

As many of the specimens, from which we generated sequences, had not been previously identified, we needed to find out, which specimens belonged in *Phyllopsora* s. str. and consequently, which sequences to use in the final phylogenetic analyses. Hence, we phylogenetically analysed a combined alignment of our Ramalinaceae dataset (Kistenich et al. 2018b) and the newly generated sequences using standard RAxML (i.e., applying the GTR substitution model for each pre-defined partition [mtSSU, ITS1, 5.8S and ITS2] with 100 rapid bootstrap inferences and the GAMMA model for evaluating and optimizing the likelihood of the final tree; Stamatakis 2014). Based on these RAxML trees, we selected those specimens falling into *Phyllopsora* s. str. and incorporated them into our *Phyllopsora* dataset (Kistenich et al. in press). This dataset was analysed phylogenetically in more detail (see below) to provide evidence for undescribed species.

Each marker was aligned separately using MAFFT v.7.408 (Katoh and Standley 2013) with the E-INS-i algorithm and the nucleotide scoring matrix set to 1PAM / κ=2. We trimmed the ends of the ITS alignment to comprise only the ITS-region and deleted the residual 18S and 28S sequence information. Each dataset was initially analysed by IQ-TREE v.1.6.7 (Nguyen et al. 2015) to infer a maximum likelihood tree using 1000 ultrafast bootstrap repetitions (Hoang et al. 2018). We checked for gene-tree incongruence using compat.py (Kauff and Lutzoni 2002) with a cut-off of 90. As we did not find any strongly supported incongruences, which would affect the circumscription of the new species, we concatenated the mtSSU and ITS alignments. We ran a detailed IQ-TREE analysis to find the best-fitting nucleotide substitution models and partitioning schemes (Chernomor et al. 2016; Kalyaanamoorthy et al. 2017) among models implemented in MrBayes (i.e., 1-, 2-, and 6-rate models) and to infer a maximum likelihood tree using 1000 standard non-parametric bootstrap repetitions (BS). We defined four subsets, one for mtSSU and three for ITS corresponding to the ITS1, 5.8S and ITS2 regions, and analysed those with the TESTMERGE function resembling PartitionFinder2. In addition, we analysed the dataset with MrBayes v.3.2.6 (Altekar et al. 2004; Ronquist and Huelsenbeck 2003) as described in Kistenich et al. (2018b). The temperature increment parameter was set to 0.05. We projected the BS values from the IQ-TREE analysis onto the MrBayes consensus tree with posterior probabilities (PP) and collapsed branches with BS < 50 and PP < 0.7. The resulting trees were edited in TreeGraph2 (Stöver and Müller 2010) and FigTree v.1.4.4 (http://tree.bio.ed.ac.uk/software/figtree).

## Results

### Morphology and secondary chemistry

Morphological identification of many specimens was challenging, but with data obtained by TLC, many specimens could be identified to species level. Of the 908 studied specimens, we found 625 specimens to belong in *Phyllopsora*, while 283 specimens were found to belong in other genera of the Malmideaceae and Ramalinaceae (not treated in this study). Of the 625 *Phyllopsora* specimens, 480 were identified to species level in *Phyllopsora* (Table 2, Suppl. material 2: Table S1), while 141 specimens (23%) were left unidentified (not included in Suppl. material 2: Table S1), most of which were not sequenced and did not contain lichen substances. The morphology and anatomy of the *Phyllopsora* species have been described in detail by Swinscow and Krog (1981) and Brako (1991), and are not repeated here. We often found the distinction between cortex type 1 and type 2 useful for species identification; however, in many species the cortex type is intermediate (type 1–2). The chemistry of the 54 accepted *Phyllopsora* species is summarized in Kistenich et al. (in press).

Information about all *Phyllopsora* species may also be found on our *Phyllopsora* website: http://nhm2.uio.no/lichens/Phyllopsora.

**Table 2.** Specimens used in this study with voucher information and GenBank accession numbers. New sequences are indicated by accession numbers in bold. – indicates missing data.

Species	Extract #	mtSSU	ITS	Country	Year	Voucher	Herbarium
*Biatorabeckhausii* (Körb.) Tuck.	–	MG925858	AF282071	Norway	1995	Holien, H. 6744	TRH
*B.vacciniicola* (Tønsberg) Printzen	–	MG925861	MG925960	Norway	2013	Klepsland, J. JK13-L330	O
*Crocyniamolliuscula* (Nyl.) Nyl.	7359	MK352275	–	La Réunion	1996	Krog, H. & Timdal, E. RE18/03	O
	7360	MK352276	–	Mauritius	1991	Krog, H. & Timdal, E. MAU58/02	O
*Phyllopsoraafricana* Timdal & Krog ch1	470	**MK412413**	**MK412480**	Thailand	1993	Aguirre, James & Wolseley 2475a	BM
	471	**MK412414**	**MK412481**	Thailand	1992	Aguirre–Hudson, B. & Wolseley, P.A. 1327	BM
	509	MK352138	MK352317	La Réunion	1996	Krog, H. & Timdal, E. RE08/13	O
	1436	MK352175	MK352348	La Réunion	1996	Krog, H. & Timdal, E. RE22/09	O
	4037	MK352199	MK352370	Thailand	2012	v.d. Boom, P. 46982	hb. v.d. Boom
	7224	**MK412469**	**MK412512**	Sri Lanka	2017	Kistenich S. & Weerakoon, G. SK1-517	PDA
*P.africana* ch1?	1012	**MK412425**	–	Indonesia	2000	Wolseley, P. T15	BM
*P.africana* ch2	477	MK352122	MK352301	Japan	1995	Thor, G. 13199	UPS
	6770	**MK412461**	**MK412504**	Sri Lanka	2017	Weerakoon, G. Ri056	PDA
*P.africana* ch3	472	**MK412415**	–	Solomon Islands	1965	Hill, D.J. 9242	BM
	1416	**MK412435**	–	Malaysia	2012	Wolseley, P., Thüs, H. & Vairappan, C. D.8.04.oQ	BORH
	1427	**MK412443**	–	Indonesia	2000	Wolseley, P. T22 OQ	BM
	6348	MK352231	MK352401	Philippines	1994	Diederich, P. 13345	hb. Diederich
	6351	**MK412447**	–	Philippines	1994	Diederich, P. 13213	hb. Diederich
	6352	**MK412448**	–	Philippines	1994	Diederich, P. 13119	hb. Diederich
	6772	**MK412462**	**MK412505**	Sri Lanka	2017	Weerakoon, G. Im015	PDA
	7205	**MK412463**	**MK412506**	Sri Lanka	2017	Kistenich S. & Weerakoon, G. SK1-543	PDA
*P.amazonica* Kistenich & Timdal	3619	MK352194	MK352365	Brazil	2014	Barbosa, R.S., Haugan, R. & Timdal, E. 90	O
	4155	MK352208	MK352379	Brazil	2015	Kistenich, S. & Timdal, E. SK1-85	MPEG
*P.breviuscula* (Nyl.) Müll. Arg.	528	MG925892	MG925990	La Réunion	1996	Krog, H. & Timdal, E. RE36/18	O
	1305	MG925893	MG925991	Brazil	1980	Kalb, K. & Marcelli, M. in: Kalb, Lich. Neotropici 515	GZU
	1432	**MK412445**	–	Sri Lanka	2007	Jayalal, U. A4-5-8-5	PDA
	2100	–	MK352355	Philippines	1992	Tan, B.C. 92-187	B
	6752	MK352245	MK352412	New Caledonia	2016	Rikkinen, J. 35509	H
	6754	**MK412456**	**MK412499**	New Caledonia	2016	Rikkinen, J. 35503	H
	6760	**MK412457**	**MK412500**	Sri Lanka	2017	Weerakoon, G. Im042	PDA
* P. breviuscula *	6764	**MK412458**	**MK412501**	Sri Lanka	2017	Weerakoon, G. Mn093	PDA
	6765	**MK412459**	**MK412502**	Sri Lanka	2017	Weerakoon, G. Mo81	PDA
	7212	**MK352256**	**MK352422**	Sri Lanka	2017	Kistenich, S. & Weerakoon, G. SK1-642	PDA
	7213	**MK412465**	**MK412508**	Sri Lanka	2017	Kistenich S. & Weerakoon, G. SK1-601	PDA
	7217	**MK412466**	**MK412509**	Sri Lanka	2017	Weerakoon, G. 982	PDA
	7218	**MK412467**	**MK412510**	Sri Lanka	2017	Weerakoon, G. 1013	PDA
	7229	**MK412470**	**MK412513**	Sri Lanka	2017	Kistenich S. & Weerakoon, G. SK1-649	PDA
	7234	–	**MK412516**	Sri Lanka	2017	Kistenich S. & Weerakoon, G. SK1-648	PDA
	7235	**MK412472**	**MK412517**	Sri Lanka	2017	Kistenich S. & Weerakoon, G. SK1-640	PDA
*P.buettneri* (Müll. Arg.) Zahlbr. ch1	428	MK352103	MK352283	Thailand	1994	Wolseley, P. & Kanajriavanit, S. s.n.	BM:734816
	995	**MK352146**	**MK352322**	Thailand	1993	James, P.W. & Wolseley, P.A. 2466a	BM
	1041	MK352160	MK352335	Kenya	2007	Divakar, Lumbsch & Mangold 19553D	hb. Pérez-Ortega
*P.buettneri* ch2	6464	MK352239	MK352406	Brazil	2015	Dahl, M.S., Kistenich, S., Timdal, E. & Toreskaas, A.K. AM-37	O
	7177	MK352252	–	Venezuela	1984	Brako, L. 8110	GZU
*P.buettneri* ch3	429	MK352104	MK352284	Thailand	1993	Aguirre, B., James, P.W. & Wolseley, P. 2736	BM
	493	**MK352131**	**MK352311**	Thailand	1994	Wolseley, P. & Kanajriavanit, S. s.n.	BM:1104011
	6462	MK352238	–	Japan	1995	Thor, G. 13183	UPS
*P.byssiseda* (Nyl.) Zahlbr.	4737	MK352211	MK352382	Venezuela	2015	Dahl, M.S., Kistenich, S., Timdal, E. & Toreskaas, A.K. SK1-220	VEN
	4739	MK352212	MK352383	Venezuela	2015	Dahl, M.S., Kistenich, S., Timdal, E. & Toreskaas, A.K. SK1-229	VEN
*P.canoumbrina* (Vain.) Brako	3627	MK352195	MK352366	Brazil	2014	Barbosa, R.S., Haugan, R. & Timdal, E. 166	O
*P.castaneocincta* (Hue) Kistenich & Timdal	460	MK352116	MK352295	Tanzania	2008	Timdal, E. 10912	O
	461	**MK412412**	**MK412479**	Thailand	1993	Aguirre, James & Wolseley 2482B	BM
	998	**MK412420**	–	Thailand	1991	Wolseley, P.A. & Aguirre–Hudson, B. 5564	BM
	999	**MK412421**	**MK412486**	Thailand	1993	Wolseley, P.A. & David, F. 3314	BM
	1022	**MK412427**	**MK412490**	Thailand	1992	Wolseley, P.A. & Aguirre–Hudson, B. 5583	BM
* P. castaneocincta *	1032	**MK412429**	–	Nepal	2007	Sharma, L.R., Olley, L., Cross L7.1	E
	1045	**MK412431**	–	Thailand	1993	James, P.W. & Wolseley, P.A. 2466b	BM
	1264	**MK412433**	**MK412492**	Malaysia	2012	Wolseley, P., Thüs, H. & Vairappan, C. M.3.10.1	BORH
	1420	**MK412439**	–	Malaysia	2012	Wolseley, P., Thüs, H. & Vairappan, C. M.3.10.2a	BORH
	1421	**MK412440**	**MK412493**	Malaysia	2012	Thüs, H., Wolseley, P. & Vairappan, C. M110	BORH
	3560	MK352186	MK352358	South Africa	2014	Burrows, J. & Timdal, E. 14280	O
	4032	MK352196	MK352367	Thailand	2012	v.d. Boom, P. 47239	hb. v.d. Boom
	6743	MK352243	MK352410	Kenya	2013	Kirika, P., Mugambi, G. & Lumbsch, H.T. 3011	O
	7232	–	**MK412515**	Sri Lanka	2017	Kistenich S. & Weerakoon, G. SK1-594	PDA
	7255	MK352270	MK352434	Australia	1992	Elix, J.A. 32834	CANB
*P.chlorophaea* (Müll. Arg.) Zahlbr.	529	MK352145	MK352321	La Réunion	1996	Krog, H. & Timdal, E. RE36/17	O
	1051	MK352165	MK352340	Kenya	2002	Killmann, D. & Fischer, E. s.n.	hb. Killmann
	1309	MK352172	–	Venezuela	1986	Brako, L. & Berry, P.E. 8685	GZU
	SE382	MG925894	MG925992	La Réunion	1996	Krog, H. & Timdal, E. RE08/10	O
*P.chodatinica* Elix	513	MK352139	–	Australia	1986	Elix, J.A. & Streimann, H. 21023	O
	1539	MK352177	MK352350	New Caledonia	2005	Elvebakk, A. 05:691	O
	6456	MK352237	MK352405	Malaysia	2014	Paukov, A. 2232	B
*P.cinchonarum* (Fée) Timdal	439	MK352105	–	Thailand	2002	Sipman, H. 48664	B
	440	MK352106	MK352285	Japan	2006	Thor, G. 21521	UPS
	4168	MK352210	MK352381	Venezuela	2015	Dahl, M.S., Kistenich, S., Timdal, E. & Toreskaas, A.K. SK1-201	VEN
	6063	MK352227	–	Guatemala	2004	v.d. Boom, P. 33395	hb. v.d. Boom
*P.concinna* Kistenich & Timdal	4041	MK352202	MK352373	Panama	2010	v.d. Boom, P. 43947	hb. v.d. Boom
	4776	MK352224	MK352395	Brazil	2015	Dahl, M.S., Kistenich, S., Timdal, E. & Toreskaas, A.K. SK1-445	O
	6455	MK352236	MK352404	Venezuela	2015	M.S. Dahl, J.E. Hernández M., S. Kistenich, E. Timdal & A.K. Toreskaas SK1-225	O
	7176	MK352251	MK352418	Guatemala	2002	Andersohn, C. s.n.	B
*P.confusa* Swinscow & Krog	514	MK352140	MK352318	Kenya	1972	Krog, H. & Swinscow, T.D.V. K48/177	O
	1018	**MK412426**	**MK412489**	Thailand	1991	Wolseley, P.A. 1049	BM
* P. confusa *	1024	MK352150	MK352325	Cuba	2007	Tønsberg, T. 37813	BG
	1300	MK352169	MK352343	Venezuela	1969	Oberwinkler, B., Oberwinkler, F. & Poelt, J. s.n.	GZU
	1417	**MK412436**	–	Malaysia	2012	Wolseley, P., Thüs, H. & Vairappan, C. M.3.10.6	BORH
	3571	MK352190	MK352362	Ecuador	2014	Prieto, M. s.n.	HUTPL
	4741	MK352214	MK352385	Venezuela	2015	Dahl, M.S., Kistenich, S., Timdal, E. & Toreskaas, A.K. SK1-237	VEN
	6360	**MK412451**	–	Papua New Guinea	1992	Diederich, P. 11056	hb. Diederich
	6361	**MK412452**	–	Papua New Guinea	1992	Diederich, P. 10319	hb. Diederich
	6766	**MK412460**	**MK412503**	Sri Lanka	2017	Weerakoon, G. Ri030	PDA
	7185	MK352253	MK352419	Cameroon	1999	Frisch, A. & Tamnjong Idi 99/Ka1213	hb. Frisch
	7220	**MK412468**	**MK412511**	Sri Lanka	2017	Weerakoon, G. 176	PDA
	7236	**MK352260**	**MK352426**	Sri Lanka	2017	Kistenich, S. & Weerakoon, G. SK1-609	PDA
	7239	**MK412473**	**MK412518**	Sri Lanka	2017	Kistenich S. & Weerakoon, G. SK1-567	PDA
	7240	**MK412474**	–	Sri Lanka	2017	Kistenich S. & Weerakoon, G. SK1-532	PDA
*P.corallina* (Eschw.) Müll. Arg.	1316	MK352173	MK352346	Venezuela	1986	Brako, L. & Berry, P.E. 8659	GZU
	4164	MK352209	MK352380	Venezuela	2015	Dahl, M.S., Kistenich, S., Timdal, E. & Toreskaas, A.K. SK1-185	VEN
	4762	MK352220	MK352391	Brazil	2015	Dahl, M.S., Kistenich, S., Timdal, E. & Toreskaas, A.K. SK1-377	O
	4775	MK352223	MK352394	Brazil	2015	Dahl, M.S., Kistenich, S., Timdal, E. & Toreskaas, A.K. SK1-430	O
*P.cuyabensis* (Malme) Zahlbr.	449	MK352107	MK352286	Peru	2006	Timdal, E. 10258	O
	450	**MK352108**	**MK352287**	Thailand	1993	Aguirre, B., James, P.W. & Wolseley, P. 2467a	BM
	1290	MK352166	MK352341	Venezuela	1996	Hafellner, J. 53910	GZU
	1291	MK352167	MK352342	Guatemala	1979	Kalb, K. & Plöbst, G. s.n.	GZU
	2048	MK352180	MK352352	Bolivia	2008	Flakus, A. & Rodriguez, P. 12792	O
*P.dolichospora* Timdal & Krog	515	MK352141	MK352319	Mauritius	1991	Krog, H. & Timdal, E. MAU65/22	O
	6357	MK352233	–	Papua New Guinea	1992	Diederich, P. 10847	hb. Diederich
* P. dolichospora *	6359	**MK412450**	–	Papua New Guinea	1992	Diederich, P. 10846	hb. Diederich
	6763	**MK352247**	**MK352414**	Sri Lanka	2017	Weerakoon, G. Hg40	PDA
	6767	**MK352248**	**MK352415**	Sri Lanka	2017	Weerakoon, G. Si113B	PDA
	7258	**MK352271**	**MK352435**	Sri Lanka	2017	Kistenich, S. & Weerakoon, G. SK1-643	PDA
*P.fendleri* (Tuck. & Mont.) Müll. Arg.	2098	MK352183	MK352354	Costa Rica	1985	H. Sipman & A. Chaverri 20806	B
	7473	MK352277	MK352437	Venezuela	1979	Sipman, H. 10688	B
*P.foliata* (Stirt.) Zahlbr.	1035	MK352157	MK352332	Japan	2004	Kashawadani, H. 46389	TNS
	7238	**MK352261**	**MK352427**	Sri Lanka	2017	Kistenich, S. & Weerakoon, G. SK1-627	PDA
	7247	MK352265	MK352431	Australia	2006	Elix, J.A. 38235	CANB
*P.foliatella* Elix	7243	MK352262	MK352428	Australia	1986	Elix, J.A. & Streimann, H. 20241	CANB
	7246	MK352264	MK352430	Australia	1986	Elix, J.A. & Streimann, H. 20203	CANB
	7253	MK352268	–	Australia	2005	Elix, J.A. 37286	CANB
	7254	MK352269	–	Australia	1998	Streimann, H. 61609	CANB
*P.furfuracea* (Pers.) Zahlbr.	451	**MK412411**	**MK412478**	Thailand	1993	Aguirre, James & Wolseley 2918	BM
	452	MK352109	MK352288	La Réunion	1996	Krog, H. & Timdal, E. RE36/22	O
	453	MK352110	MK352289	Trinidad And Tobago	2008	Rui, S. & Timdal, E. 10799	O
	455	MK352111	MK352290	Peru	2006	Timdal, E. 10183	O
*P.furfurella* Kistenich & Timdal	3570	MK352189	MK352361	Ecuador	2014	Prieto, M. s.n.	HUTPL
	4036	MK352198	MK352369	Dominican Republic	2008	v.d. Boom, P. 39069	hb. v.d. Boom
*P.glaucella* (Vain.) Timdal	1000	MK352147	MK352323	Dominican Republic	1987	Harris, R.C. 20779	BM
	2125	MK352184	MK352356	Argentina	2013	Ferraro, L.I., Aptroot, A. & Cáceres, M.E.S. 10761	O
	4766	MK352221	MK352392	Brazil	2015	Dahl, M.S., Kistenich, S., Timdal, E. & Toreskaas, A.K. SK1-393	O
	4780	MK352225	MK352396	Brazil	2015	Dahl, M.S., Kistenich, S., Timdal, E. & Toreskaas, A.K. AM-44	O
*P.gossypina* (Sw.) Kistenich et al.	–	AY584615	–	Costa Rica	2002	Lücking, R. 16052	DUKE
*P.gossypina* ch1	3575	MK352192	MK352363	Brazil	2014	Barbosa, R.S., Haugan, R. & Timdal, E. 141	O
	3576	MK352193	MK352364	Brazil	2014	Barbosa, R.S., Haugan, R. & Timdal, E. 34	O
	4160	MG925867	MG925967	Brazil	2015	Kistenich, S. & Timdal, E. SK1-108	O
*P.gossypina* ch1	4746	MG925868	MG925968	Brazil	2015	Dahl, M.S., Kistenich, S., Timdal, E. & Toreskaas, A.K. SK1-287	O
	7201	**MK352254**	**MK352420**	Sri Lanka	2017	Kistenich, S. & Weerakoon, G. SK1-584	PDA
*P.gossypina* ch2	4750	MK352219	MK352390	Brazil	2015	Dahl, M.S., Kistenich, S., Timdal, E. & Toreskaas, A.K. SK1-297	O
*P.halei* (Tuck.) Zahlbr. ch2	457	MK352113	MK352292	Tanzania	2008	Timdal, E. 10931	O
	1044	MK352161	MK352336	Kenya	2007	Divakar, Lumbsch & Mangold 19574K	hb. Pérez–Ortega
*P.halei* ch3	7221	**MK352257**	**MK352423**	Sri Lanka	2017	Weerakoon, G. 1008	PDA
*P.hispaniolae* Timdal	1545	MK352178	–	Ecuador	1999	Palice, Z. 3875	hb. Palice
	3569	MK352188	MK352360	Ecuador	2014	Prieto, M. s.n.	HUTPL
	4039	MK352201	MK352372	Panama	2010	v.d. Boom, P. 44158	hb. v.d. Boom
*P.imshaugii* Timdal	3558	MK352185	MK352357	Ecuador	2014	Prieto, M. s.n.	HUTPL
	4043	MK352204	MK352375	Guatemala	2004	v.d. Boom, P. 33433	hb. v.d. Boom
	4744	MK352217	MK352388	Venezuela	2015	Dahl, M.S., Kistenich, S., Timdal, E. & Toreskaas, A.K. SK1-253	VEN
*P.isidiosa* Kistenich & Timdal	430	**MK412409**	**MK412476**	Thailand	1991	Wolseley, P.A. & Aguirre–Hudson, B. 5552	BM
	1027	MK352153	MK352328	USA	2006	Lendemer, J.C. 7765 dupl.	BG
	1030	MK352155	MK352330	Nepal	2007	Sharma, L.R., Olley, L., Cross, A., Joshi, M. & Regmi, B. M16	E
	1031	**MK412428**	–	Nepal	2007	Sharma, L.R., Olley, L., Cross L25-2	E
	1259	**MK412432**	–	Malaysia	2012	Wolseley, P., Thüs, H. & Vairappan, C. S.P.5	BORH
	2099	**MK412446**	**MK412494**	Indonesia	2003	L. Sudirman & H. Sipman 51474	B
	4035	MK352197	MK352368	Dominican Republic	2008	v.d. Boom, P. 39012	hb. v.d. Boom
	4781	MG925907	MG926004	Brazil	2007	Lücking, R & Rivas Plata, E. 23302	SP
	6349	MK352232	–	Philippines	1994	Diederich, P. 13210	hb. Diederich
	7251	MK352267	MK352433	Australia	2006	Elix, J.A. 38478	CANB
*P.isidiotyla* (Vain.) Riddle	1315	MG925906	MG926003	Brazil	1979	Kalb, K. & Plöbst, G. in: Kalb, Lich. Neotrop. 343	GZU
*P.kalbii* Brako	456	**MK352112**	**MK352291**	Thailand	1993	Aguirre, B., James, P.W. & Wolseley, P. 2695	BM
	458	MK352114	MK352293	Tanzania	2008	Timdal, E. 10913	O
	459	MK352115	MK352294	Venezuela	1989	Kalb, K. & A. s.n.	O
	1028	MK352154	MK352329	USA	2010	Lendemer, J.C. 25770	BG
	2052	MK352182	–	Bolivia	2010	Flakus, A. & Quisbert, J. 19221	O
*P.loekoesii* S.Y. Kondr. et al.	1033	MK352156	MK352331	Nepal	2007	Sharma, L.R., Olley, L., Cross A. C5	E
	7478	MK352279	MK352439	Japan	1994	Thor, G. 12574	TNS
*P.longiuscula* (Nyl.) Zahlbr.	454	MG925899	MG925996	Peru	2006	Timdal, E. 10433	O
	467	MK352117	MK352296	Trinidad And Tobago	2008	Rui, S. & Timdal, E. 10730	O
	1011	**MK412424**	**MK412488**	Thailand	1992	Wolseley, P.A. & Aguirre–Hudson, B. 5580 p.p.	BM
	1039	MK352159	MK352334	Cuba	2006	Pérez–Ortega, S. s.n.	hb. Pérez–Ortega
	6761	**MK352159**	**MK352413**	Sri Lanka	2017	Weerakoon, G. Kn136	PDA
*P.malcolmii* Vezda & Kalb	1303	MK352170	MK352344	New Zealand	1994	Malcolm, W. in: Vezda, Lich. Rar. Exs. 200	GZU
*P.martinii* Swinscow & Krog	489	MK352129	MK352309	Tanzania	1989	Krog, H. 3T13/007	O
	6740	MK352242	MK352409	Kenya	2014	Kirika, P. & Lumbsch, H.T. 4087	O
*P.mauritiana* (Taylor) Swinscow & Krog	487	MK352128	MK352307	Tanzania	1988	Krog, H. 2T12/037	O
	488	–	MK352308	Mauritius	1991	Krog, H. & Timdal, E. MAU09/43	O
	SE386	MG925900	MG925997	Mauritius	1991	Krog, H. & Timdal, E. MAU09/44	O
*P.mediocris* Swinscow & Krog	527	MK352144	MK352320	Tanzania	1988	Krog, H. 2T06/023	O
	6346	MK352229	MK352399	Mauritius	2016	Diederich, P. 18571	hb. Diederich
	6347	MK352230	MK352400	Mauritius	2016	Diederich, P. 18573	hb. Diederich
*P.melanoglauca* Zahlbr.	1038	MK352158	MK352333	Cuba	2006	Pérez–Ortega, S. s.n.	hb. Pérez–Ortega
	4042	MK352203	MK352374	Guatemala	2004	v.d. Boom, P. 33408	hb. v.d. Boom
	4740	MK352213	MK352384	Venezuela	2015	Dahl, M.S., Kistenich, S., Timdal, E. & Toreskaas, A.K. SK1-232	VEN
	4743	MK352216	MK352387	Venezuela	2015	Dahl, M.S., Kistenich, S., Timdal, E. & Toreskaas, A.K. SK1-247	VEN
	6450	MK352235	MK352403	Brazil	2015	Dahl, M.S., Kistenich, S., Timdal, E. & Toreskaas, A.K. SK1-408	O
*P.nemoralis* Timdal & Krog	522	MK352142	–	La Réunion	1996	Krog, H. & Timdal, E. RE25/32	O
	1434	MK352174	MK352347	South Africa	1996	Nordin, A. 4622	UPS:L:92604
*P.neofoliata* Elix	6745	MK352244	MK352411	Kenya	2015	Kirika, P. & Lumbsch, H.T. 4728	O
	7245	MK352263	MK352429	Australia	1992	Elix, J.A. 32714	O
	7249	MK352266	MK352432	Australia	1989	Elix, J.A.	CANB
*P.neotinica* Kistenich & Timdal	505	MK352137	MK352316	Trinidad And Tobago	2008	Rui, S. & Timdal, E. 10774	O
	1023	MK352149	MK352324	Cuba	2007	Tønsberg, T. 37923	BG
	1438	MK352176	MK352349	Trinidad And Tobago	2008	Rui, S. & Timdal, E. 10763	O
* P. neotinica *	4742	MK352215	MK352386	Venezuela	2015	M.S. Dahl, J.E. Hernández M., S. Kistenich, E. Timdal & A.K. Toreskaas SK1-246	O
	4769	MK352222	MK352393	Brazil	2015	Dahl, M.S., Kistenich, S., Timdal, E. & Toreskaas, A.K. SK1-402	O
*P.ochroxantha* (Nyl.) Zahlbr.	473	MK352118	MK352297	Peru	2006	Timdal, E. 10338	O
	474	MK352119	MK352298	Peru	2006	Timdal, E. 10389	O
	475	MK352120	MK352299	Trinidad And Tobago	2008	Rui, S. & Timdal, E. 10849	O
	4049	MK352206	MK352377	Brazil	2015	Kistenich, S. & Timdal, E. SK1-47	O
	4747	MK352218	MK352389	Brazil	2015	Dahl, M.S., Kistenich, S., Timdal, E. & Toreskaas, A.K. SK1-289	O
*P.parvifolia* (Pers.) Müll. Arg.	479	MK352124	MK352303	Tanzania	2008	Timdal, E. 10935	O
	480	MK352125	MK352304	Trinidad And Tobago	2008	Rui, S. & Timdal, E. 10867	O
	2049	MK352181	MK352353	Bolivia	2010	Flakus, A. & Quisbert, J. 20016	O
	3561	MK352187	MK352359	South Africa	2014	Burrows, J. & Timdal, E. 14244	O
	6365	MK352234	MK352402	Portugal	2015	v.d. Boom, P. 53877	hb. v.d. Boom
*P.parvifoliella* (Nyl.) Müll. Arg.	481	MK352126	MK352305	Peru	2006	Timdal, E. 10302	O
	482	**MG925902**	**MG925999**	Indonesia	2000	Wolseley, P.A. s.n.	BM:1104069
	483	**MK352127**	**MK352306**	Thailand	1993	James, P.W. & Wolseley, P.A. 2491	BM
	1004	**MK412422**	–	Thailand	1993	James, P.W. & Wolseley, P.A. 1847	BM
*P.phaeobyssina* (Vain.) Timdal	478	MK352123	MK352302	Trinidad And Tobago	2008	Rui, S. & Timdal, E. 10872	O
*P.porphyromelaena* (Vain.) Zahlbr. ch1	490	**MK412416**	**MK412482**	Thailand	1994	Wolseley, P. & Kanajriavanit, S. s.n.	BM:1104012
	498	MG925904	MG926001	La Réunion	1996	Krog, H. & Timdal, E. RE07/17	O
	502	MK352135	MK352314	Japan	1995	Thor, G. 12941	UPS
	1050	MK352164	MK352339	Kenya	2002	Killmann, D. & Fischer, E. s.n.	hb. Killmann
	1429	**MK412444**	–	Sri Lanka	2007	Jayalal, U. B9-4-3-3	PDA
	7207	**MK412464**	**MK412507**	Sri Lanka	2017	Kistenich S. & Weerakoon, G. SK1-634	PDA
*P.porphyromelaena* ch2	491	**MK412417**	**MK412483**	Thailand	1993	Aguirre–Hudson, B. & Wolseley, P.A. 1663	BM
	496	MK352133	–	Tanzania	1989	Krog, H. 4T16/019	O
	503	MK352136	MK352315	Japan	2006	Thor, G. 21238	UPS
	6436	**MK412454**	**MK412497**	Malaysia	2014	Paukov, A. 2233	B
	7208	**MK352255**	**MK352421**	Sri Lanka	2017	Kistenich, S. & Weerakoon, G. SK1-631	PDA
*P.porphyromelaena* ch2	7479	**MK412475**	**MK412519**	Japan	2017	Haugan, R. & Timdal, E. 16753	O:L:209897
*P.porphyromelaena* ch3	492	**MK352130**	**MK352310**	Thailand	1993	Aguirre, B., James, P.W. & Wolseley, P. 2857	BM
	494	**MK352132**	**MK352312**	Thailand	1993	Aguirre, B., James, P.W. & Wolseley, P. 2481	BM
*P.pseudocorallina* Kistenich & Timdal	1034	**MK412430**	**MK412491**	Cambodia	2005	Kashiwadani, H. 47806	TNS
	1418	**MK412437**	–	Malaysia	2012	Thüs, H., Wolseley, P. & Vairappan, C. M001a	BORH
	1419	**MK412438**	–	Malaysia	2012	Thüs, H., Wolseley, P. & Vairappan, C. M005	BORH
	6356	**MK412449**	**MK412495**	Papua New Guinea	1992	Diederich, P. 11386	hb. Diederich
*P.pyxinoides* (Nyl.) Kistenich et al.	3574	MK352191	–	Brazil	2014	Cáceres, M., Haugan, R. & Timdal, E. 21024	O
	7358	MK352274	–	USA	1991	Ryan, B. 27530	O
*P.rappiana* (Brako) Elix	6737	MK352240	MK352407	Australia	2005	Elix, J. 36867	O
	7175	MK352250	MK352417	Panama	2010	v.d. Boom, P. 43820	hb. v.d. Boom
*P.rosei* Coppins & P. James	1299	MK352168	–	UK	1992	Coppins, B., James, P.W. & Poelt, J. Sc92/446	GZU
	6339	MK352228	MK352398	France	2000	Diederich, P. 14602	hb. Diederich
	7356	MK352272	MK352436	France	1990	Diederich, P. 9247	hb. Diederich
	7357	MK352273	–	UK	1992	Coppins, B., James, P.W. & Poelt, J. Sc92/193	GZU
*P.sabahana* Kistenich & Timdal	1265	**MK412434**	–	Malaysia	2012	Wolseley, P., Thüs, H. & Vairappan, C. S.B.oQ.3	BORH
	1423	**MK412441**	–	Malaysia	2012	Thüs, H., Wolseley, P. & Vairappan, C. M089	BORH
	1425	**MK412442**	–	Malaysia	2012	Wolseley, P., Thüs, H. & Vairappan, C. D.8.02.4	BORH
	6435	**MK412453**	**MK412496**	Malaysia	2014	Paukov, A. 2230	B
	6457	**MK412455**	**MK412498**	Malaysia	2014	Paukov, A. 2229	B
*P.santensis* (Tuck.) Swinscow & Krog	2043	MK352179	MK352351	Bolivia	2009	Flakus, A. & Rodriguez, P. 15581	O
	4038	MK352200	MK352371	Panama	2010	v.d. Boom, P. 44704	hb. v.d. Boom
	4051	MK352207	MK352378	Brazil	2015	Kistenich, S. & Timdal, E. SK1-79	O
*P.siamensis* Kistenich & Timdal	448	**MK412410**	**MK412477**	Thailand	1993	Wolseley, P.A. & Boonpragob, K. 3245	BM
	996	**MK412418**	**MK412484**	Thailand	1992	Wolseley, P.A. & Onsar 5590	BM
	997	**MK412419**	**MK412485**	Thailand	1993	Aguirre–Hudson, B. & Wolseley, P.A. 1643	BM
	1010	**MK412423**	**MK412487**	Thailand	1992	Wolseley, P.A. & Aguirre–Hudson, B. 5580	BM
*P.* sp. 1	7230	**MK352259**	**MK352425**	Sri Lanka	2017	Kistenich, S. & Weerakoon, G. SK1-545	PDA
*P.* sp. 2	1017	**MK352148**	–	Malaysia	1997	Wolseley, P. s.n.	BM:1104019
*P.* sp. 3	7227	**MK352258**	**MK352424**	Sri Lanka	2017	Kistenich, S. & Weerakoon, G. SK1-555	PDA
*P.* sp. 4	7231	**MK412471**	**MK412514**	Sri Lanka	2017	Kistenich S. & Weerakoon, G. SK1-570	PDA
*P.subhispidula* (Nyl.) Kalb & Elix	501	MK352134	MK352313	Tanzania	1989	Krog, H. 4T15/007	O
	6738	MK352241	MK352408	La Réunion	1996	Krog, H. & Timdal, E. RE36/15	O
	6771	**MK352249**	**MK352416**	Sri Lanka	2017	Weerakoon, G. Hg29A	PDA
*P.swinscowii* Timdal & Krog	476	MK352121	MK352300	Peru	2006	Timdal, E. 10190	O
	525	MK352143	–	Mauritius	1991	Krog, H. & Timdal, E. MAU09/50	O
	1025	MK352151	MK352326	Cuba	2007	Tønsberg, T. 37817	BG
	1049	MK352163	MK352338	Kenya	2002	Killmann, D. & Fischer, E. s.n.	hb. Killmann
	4048	MK352205	MK352376	Brazil	2015	Kistenich, S. & Timdal, E. SK1-115	O
*P.teretiuscula* Timdal	1026	MK352152	MK352327	Cuba	2007	Tønsberg, T. 37814	BG
	1306	MK352171	MK352345	Costa Rica	2003	Hafellner & Emmerer 1490	GZU
	7474	MK352278	MK352438	Puerto Rico	1992	Harris, R.C. 27320	O
*P.thaleriza* (Stirt.) Brako	1048	MK352162	MK352337	Kenya	2003	Killmann, D. & Fischer, E. s.n.	hb. Killmann
	5465	MG925880	MG925982	South Africa	2014	Burrows, J. & Timdal, E. 14191	O
	5466	MG925881	MG925983	South Africa	2015	Rui, S. & Timdal, E. 13877	O
	5467	MK352226	MK352397	South Africa	2015	Rui, S. & Timdal, E. 13873	O

### Molecular data and phylogenetic analysis

We obtained sequences for 140 phyllopsoroid specimens with 132 mtSSU and 106 ITS sequences (Tables 2, 3). Based on the initial RAxML analyses (not shown), 93 specimens were found to belong in *Phyllopsora* s. str. (Table 2) and were used in the subsequent analyses. The remaining 47 specimens did not belong in *Phyllopsora* s. str. (Table 3) and are referred to at the family level only due to many problems of generic affiliation.

The concatenated alignment had a length of 1,825 bp with 264 accessions including one specimen of *Biatorabeckhausii* (Körb.) Tuck. and one of *B.vacciniicola* (Tønsberg) Printzen for rooting of the phylogenetic trees. The alignment contained ca. 20% missing data and is available from TreeBase (study no. 23881).

**Figure 1.**
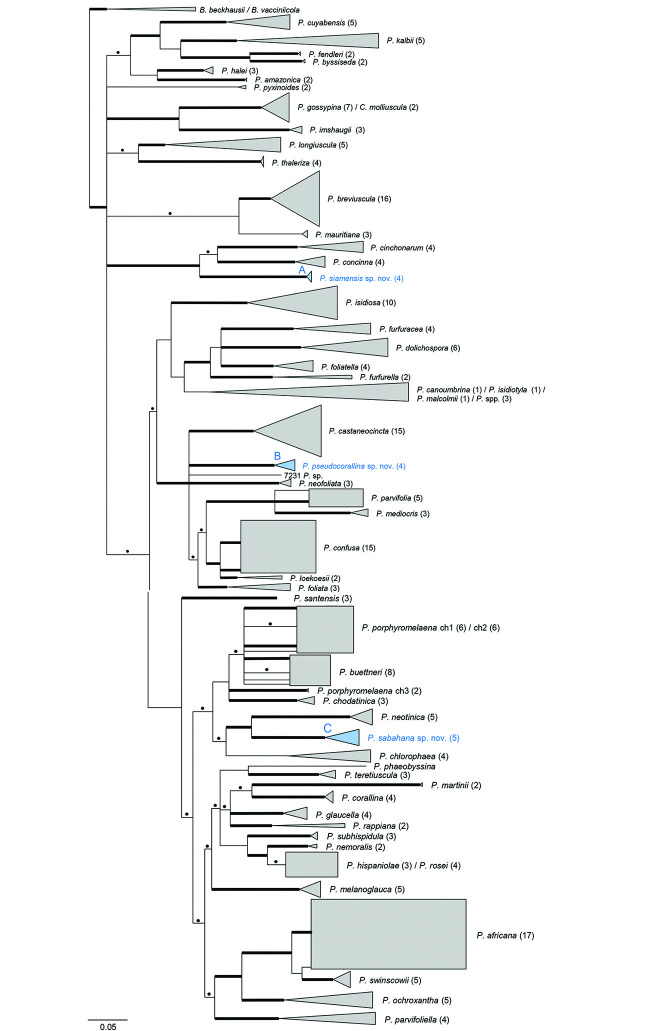
Extended majority-rule consensus tree resulting from the MrBayes analysis of the mtSSU and ITS alignment with Bayesian PP ≥ 0.7 and/or IQ-TREE maximum likelihood BS ≥ 50 and branch lengths. Strongly supported branches (PP ≥ 0.95 and BS ≥ 75) are marked in bold; branches only supported with PP ≥ 0.7 or BS ≥ 50 are marked with a dot above the branch. Two species of *Biatora* were used for rooting. Accessions belonging to the same species are collapsed for convenience. Three clades are distinguished to facilitate the discussion of new species (**A, B, C**). ch = chemotype.

The software IQ-TREE suggested the following substitution models for four subsets: GTR+I+Γ for mtSSU and SYM+I+Γ for ITS1, 5.8S and ITS2. Bayesian phylogenetic analysis halted automatically after 40×10^6^ generations, when the ASDSF in the last 50% of each run had fallen below 0.01. Following a burnin of 50%, we used 80,004 trees for the final Bayesian majority-rule consensus tree. The phylogenetic results generated by IQ-TREE vs. MrBayes showed no incongruences. The extended majority-rule consensus tree (Fig. 1; see Suppl. material 1: Fig. S1 for the uncollapsed version of the tree), based on the Bayesian topology with all compatible groups (BS ≥ 50 and/or PP ≥ 0.7), shows an overall good resolution of *Phyllopsora* species. Seventeen unidentified specimens did not associate with any known species in the phylogenetic tree (Fig. 1). Four unidentified specimens (1017, 7227, 7230, and 7231) were resolved on long branches, while the remaining 13 specimens grouped into three distinct, strongly supported clades (Fig. 1A–C). Clade A is resolved as sister to a clade consisting of *P.cinchonarum* and *P.concinna*, clade B is found in a clade with *P.castaneocincta*, *P.foliata* and *P.neofoliata* among others, and clade C is resolved as sister to *P.neotinica*.

**Table 3.** Newly generated sequences for specimens not belonging to *Phyllopsora* with voucher information and GenBank accession numbers. – indicates missing data.

Family	Extract #	mtSSU	ITS	Country	Year	Voucher	Herb.
Malmideaceae	1268	MK400188	MK400239	Malaysia	2012	Wolseley, P., Thüs, H. & Vairappan, C. D.1.10.3	BORH
Ramalinaceae	417	MK400189	MK400240	Thailand	1993	Wolseley, P.A. & David, F. 3347	BM:749829
	423	MK400190	MK400241	Indonesia	2000	Wolseley, P. T9 LQ	BM:1104053
	427	MK400191	MK400242	Indonesia	2000	Wolseley, P. T13 LQ	BM:1104062
	432	MK400192	MK400243	Malaysia	1997	Wolseley, P. pkt. 8	BM:1104016
	433	MK400193	MK400244	Thailand	1991	Wolseley, P.A. & Aguirre–Hudson, B. 5548	BM:749824
	435	MK400194	MK400245	Indonesia	2000	Wolseley, P. T20 LMQ	BM:1104013
	1008	MK400195	–	Thailand	1993	Aguirre, James & Wolseley 2854	BM
	1013	MK400196	–	Thailand	1993	James, P.W. & Wolseley, P.A. 1700b	BM
	1014	MK400197	MK400246	Thailand	1993	Aguirre, James & Wolseley 2478a	BM:749861
	1015	MK400198	MK400247	Thailand	1993	Aguirre, James & Wolseley 2715	BM:749853
	1020	MK400199	–	Indonesia	2000	Wolseley, P. T6 LQ	BM:1104066
	1021	MK400200	–	Indonesia	2000	Wolseley, P. T1	BM:1104063
	1266	–	MK400248	Malaysia	2012	Wolseley, P., Thüs, H. & Vairappan, C. D.4.04.2	BORH
	1270	MK400201	MK400249	Malaysia	2012	Wolseley, P., Thüs, H. & Vairappan, C. M.1.12.oQ	BORH
	1275	MK400202	MK400250	Malaysia	2012	Wolseley, P., Thüs, H. & Vairappan, C. D+40	BORH
	1282	MK400203	MK400251	Malaysia	2012	Wolseley, P., Thüs, H. & Vairappan, C. S.B.10.2	BORH
	1284	MK400204	MK400252	Malaysia	2012	Wolseley, P., Thüs, H. & Vairappan, C. D.7.09.1	BORH
	1285	MK400205	MK400253	Malaysia	2012	Wolseley, P., Thüs, H. & Vairappan, C. M.3.08.oQ.2	BORH
	1287	MK400206	MK400254	Malaysia	2012	Wolseley, P., Thüs, H. & Vairappan, C. M.3.03.1	BORH
	1426	–	MK400255	Malaysia	2013	Vairappan, C. L261	BM
	1428	MK400207	MK400256	Thailand	1993	Aguirre, James & Wolseley 2477e	BM:1031544
	6056	MK400208	–	Malaysia	2014	Paukov, A. 2236	B
	6057	MK400209	–	Malaysia	2014	Paukov, A. 2235	B
	6762	MK400210	MK400257	Sri Lanka	2017	Weerakoon, G. Ne141	PDA
	6768	MK400211	MK400258	Sri Lanka	2017	Weerakoon, G. WL60	PDA
	6769	MK400212	MK400259	Sri Lanka	2017	Weerakoon, G. WL15/2	PDA
	7186	MK400213	MK400260	Sri Lanka	2017	Kistenich, S. & Weerakoon, G. SK1-651	PDA
	7187	MK400214	MK400261	Sri Lanka	2017	Kistenich, S. & Weerakoon, G. SK1-650	PDA
	7188	MK400215	MK400262	Sri Lanka	2017	Kistenich, S. & Weerakoon, G. SK1-564	PDA
	7189	MK400216	–	Sri Lanka	2017	Kistenich, S. & Weerakoon, G. SK1-566	PDA
	7190	MK400217	MK400263	Sri Lanka	2017	Kistenich, S. & Weerakoon, G. SK1-604	PDA
	7191	MK400218	MK400264	Sri Lanka	2017	Kistenich, S. & Weerakoon, G. SK1-602	PDA
	7192	MK400219	MK400265	Sri Lanka	2017	Kistenich, S. & Weerakoon, G. SK1-611	PDA
	7193	MK400220	MK400266	Sri Lanka	2017	Kistenich, S. & Weerakoon, G. SK1-558	PDA
	7195	MK400221	MK400267	Sri Lanka	2017	Kistenich, S. & Weerakoon, G. SK1-560	PDA
	7196	MK400222	MK400268	Sri Lanka	2017	Kistenich, S. & Weerakoon, G. SK1-673	PDA
Ramalinaceae	7198	MK400223	MK400269	Sri Lanka	2017	Kistenich, S. & Weerakoon, G. SK1-659	PDA
	7199	MK400224	MK400270	Sri Lanka	2017	Kistenich, S. & Weerakoon, G. SK1-587	PDA
	7202	–	MK400271	Sri Lanka	2017	Kistenich, S. & Weerakoon, G. SK1-573	PDA
	7204	–	MK400272	Sri Lanka	2017	Kistenich, S. & Weerakoon, G. SK1-524	PDA
	7206	MK400225	MK400273	Sri Lanka	2017	Kistenich, S. & Weerakoon, G. SK1-666	PDA
	7211	MK400226	MK400274	Sri Lanka	2017	Kistenich, S. & Weerakoon, G. SK1-561	PDA
	7215	–	MK400275	Sri Lanka	2017	Kistenich, S. & Weerakoon, G. SK1-592	PDA
	7222	MK400227	MK400276	Sri Lanka	2017	Weerakoon, G. 641 loc.31	PDA
	7226	–	MK400277	Sri Lanka	2017	Kistenich, S. & Weerakoon, G. SK1-628	PDA
	7228	MK400228	–	Sri Lanka	2017	Kistenich, S. & Weerakoon, G. SK1-667	PDA

## Discussion

**Figure 2.**
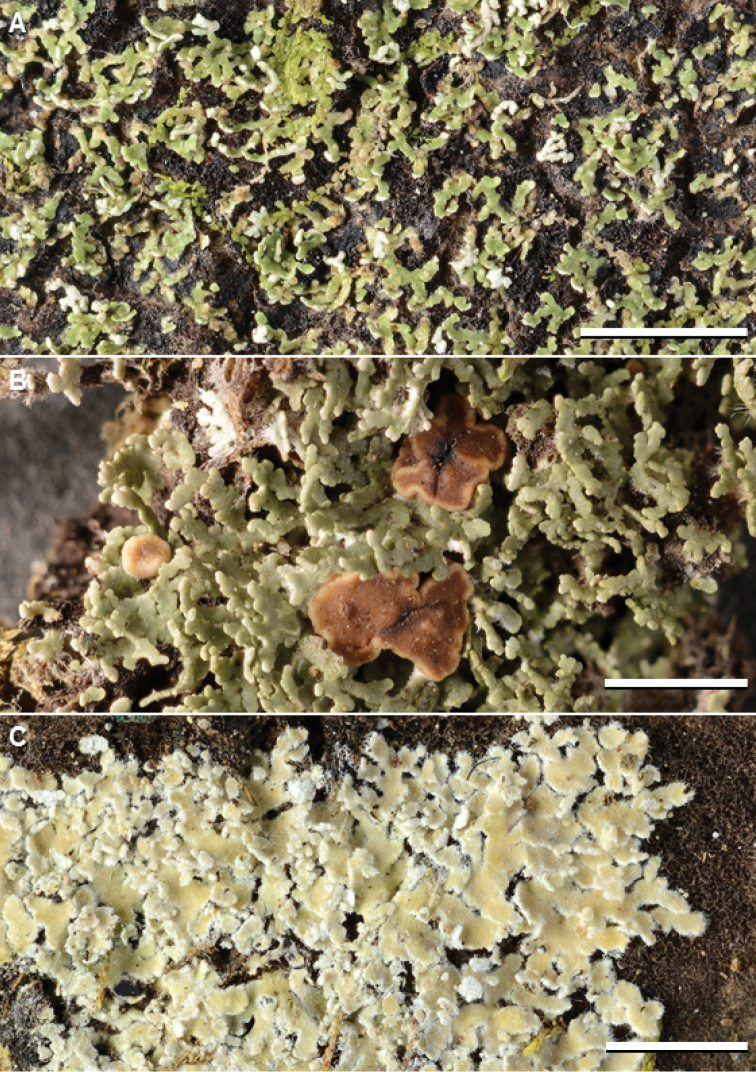
Species of *Phyllopsora* occurring in Asia and Melanesia. **A***Phyllopsoraafricana* (Kistenich & Weerakoon SK1-517) **B***P.breviuscula* (Kistenich & Weerakoon SK1-601) **C***P.buettneri* (Thor 13183). Scale bars: 2 mm.

**Figure 3.**
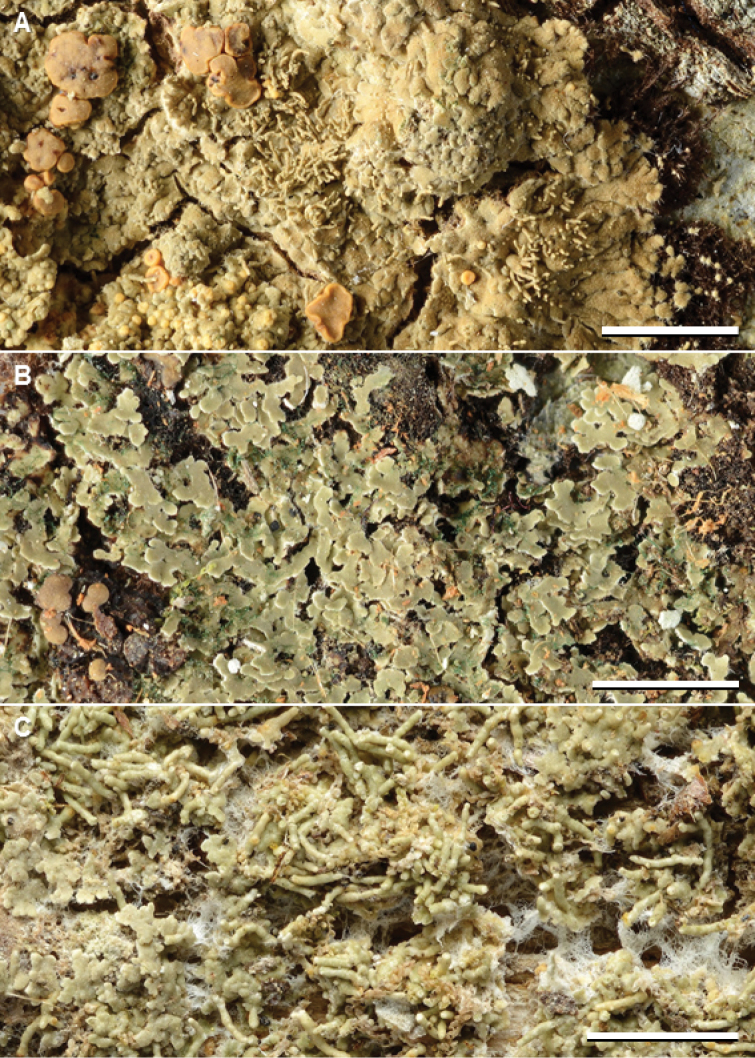
Species of *Phyllopsora* occurring in Asia and Melanesia. A *Phyllopsoracastaneocincta* (Kirika, Mugambi, & Lumbsch 3011) B *P.chodatinica* (Paukov 2232) C *P.cinchonarum* (Thor 21521). Scale bars: 2 mm.

**Figure 4.**
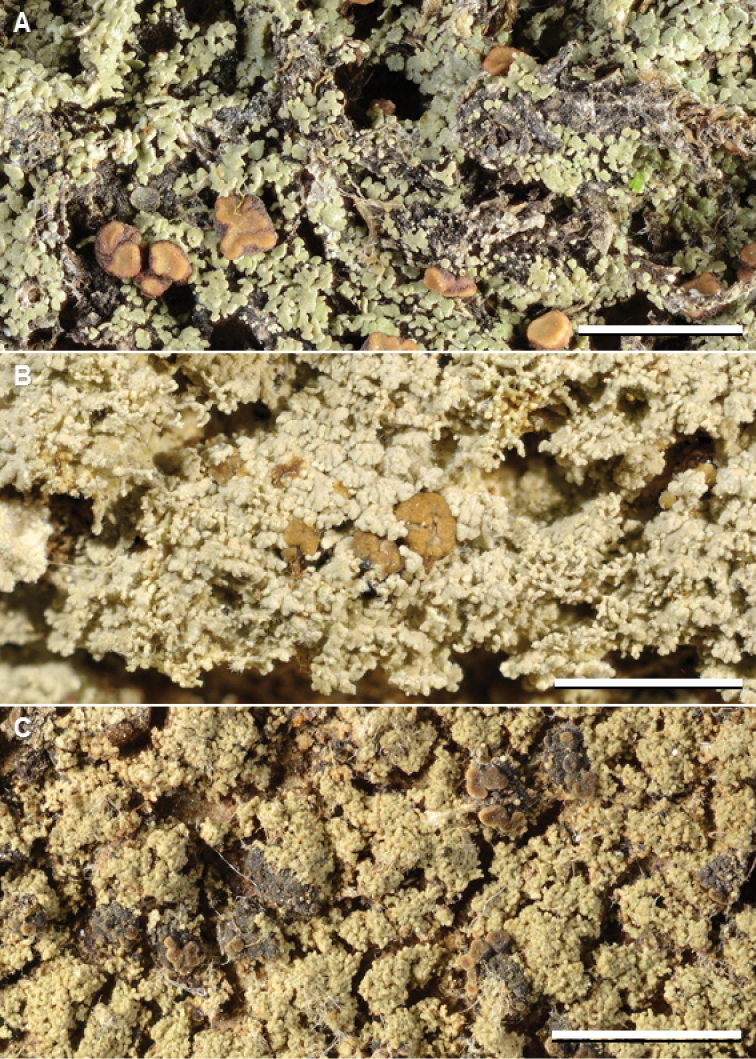
Species of *Phyllopsora* occurring in Asia and Melanesia. **A***Phyllopsoraconfusa* (Kistenich & Weerakoon SK1-532) **B***P.cuyabensis* (Aguirre, James & Wolseley 2467a) **C***P.dolichospora* (Weerakoon Si113B). Scale bars: 2 mm.

**Figure 5.**
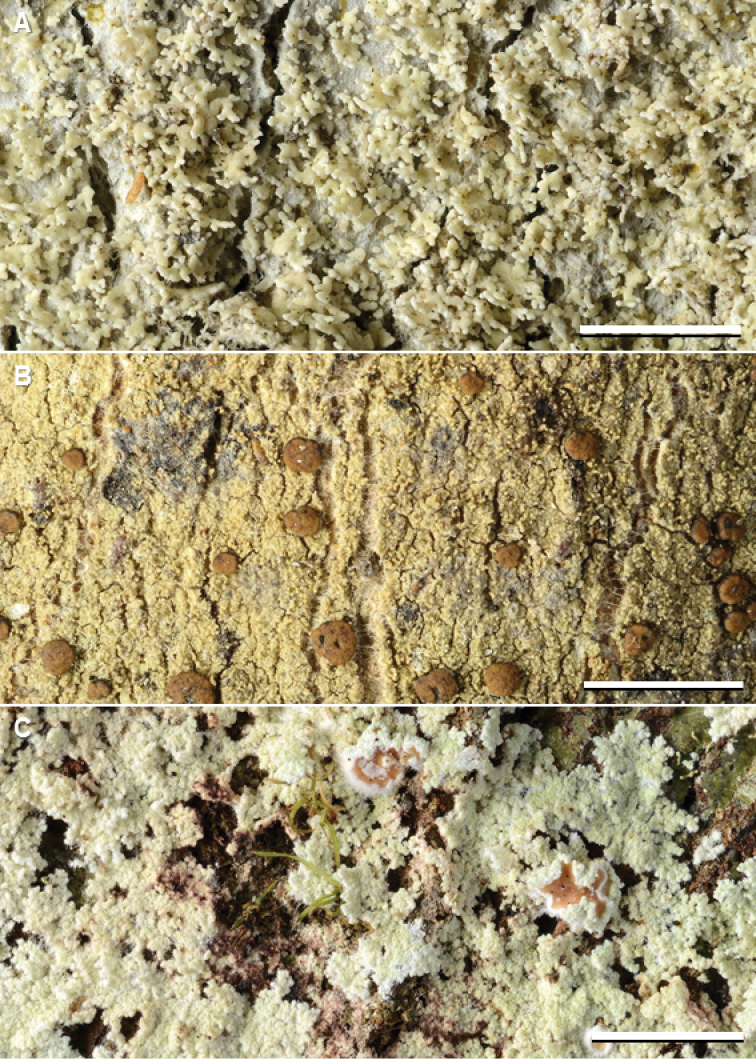
Species of *Phyllopsora* occurring in Asia and Melanesia. **A***Phyllopsorafoliata* (Kistenich & Weerakoon SK1-627) **B***P.furfuracea* (Wolseley & Aguirre-Hudson 4025) **C***P.gossypina* (Kistenich & Weerakoon SK1-524). Scale bars: 2 mm.

**Figure 6.**
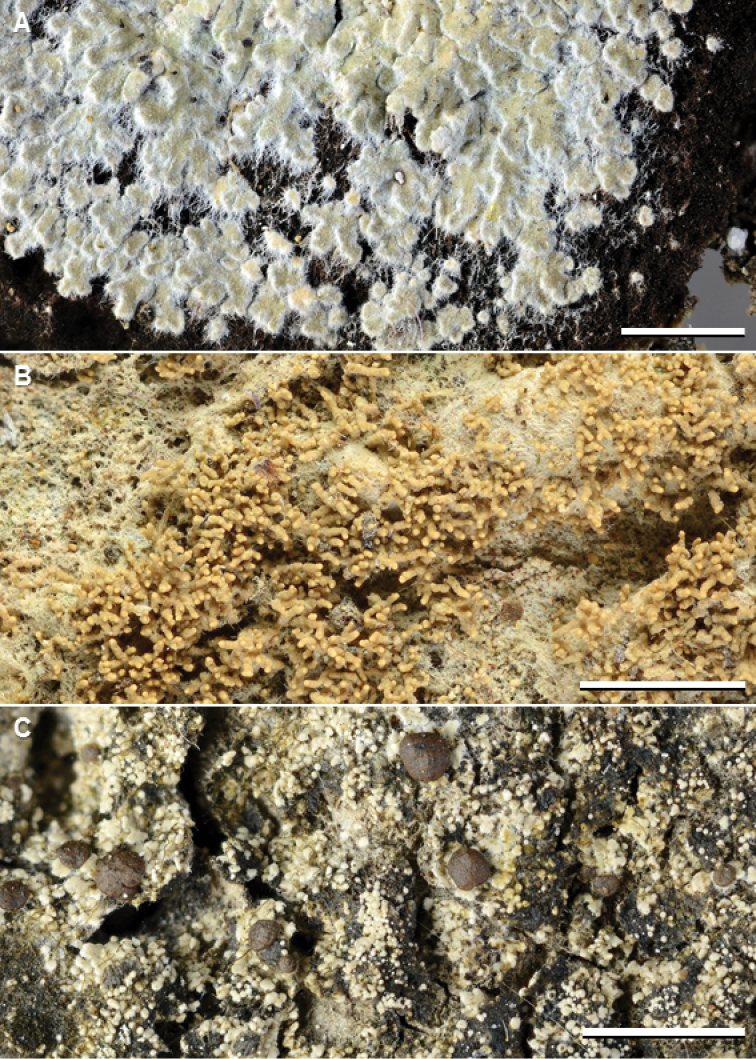
Species of *Phyllopsora* occurring in Asia and Melanesia. **A***Phyllopsorahalei* (Weerakoon 1008) **B***P.isidiosa* (Diederich 13210) **C***P.kalbii* (Aguirre, James & Wolseley 2695). Scale bars: 2 mm.

**Figure 7.**
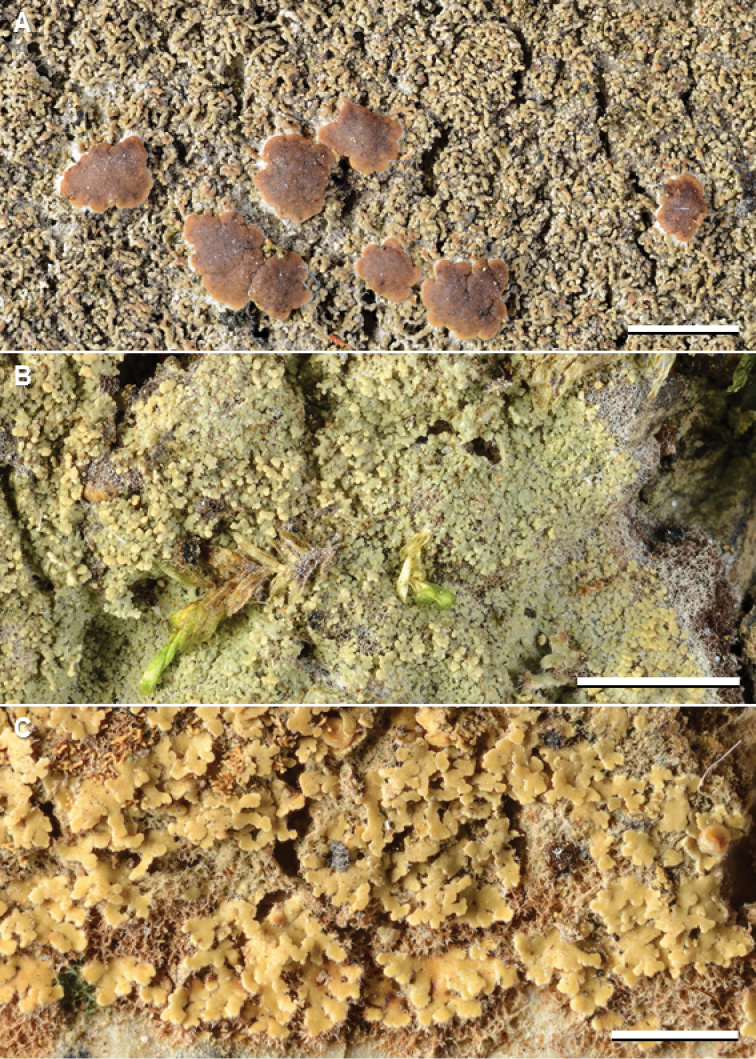
Species of *Phyllopsora* occurring in Asia and Melanesia. **A***Phyllopsoraloekoesii* (Sharma, Olley & Cross AC5) **B***P.longiuscula* (Weerakoon Kn136) **C***P.mediocris* (holotype, Moberg 1481a-1, Tanzania). Scale bars: 2 mm.

**Figure 8.**
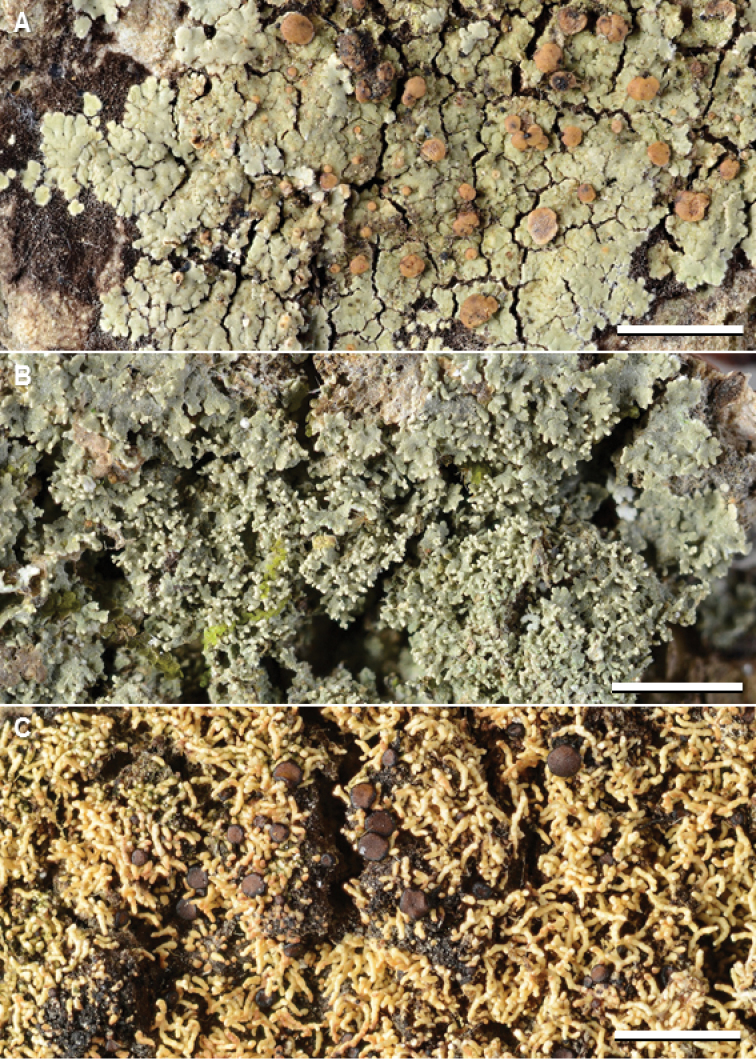
Species of *Phyllopsora* occurring in Asia and Melanesia. **A***Phyllopsoraneofoliata* (Weerakoon WL21) **B***P.parvifolia* (Kistenich & Weerakoon SK1-661) **C***P.parvifoliella* (James & Wolseley 2491). Scale bars: 2 mm.

**Figure 9.**
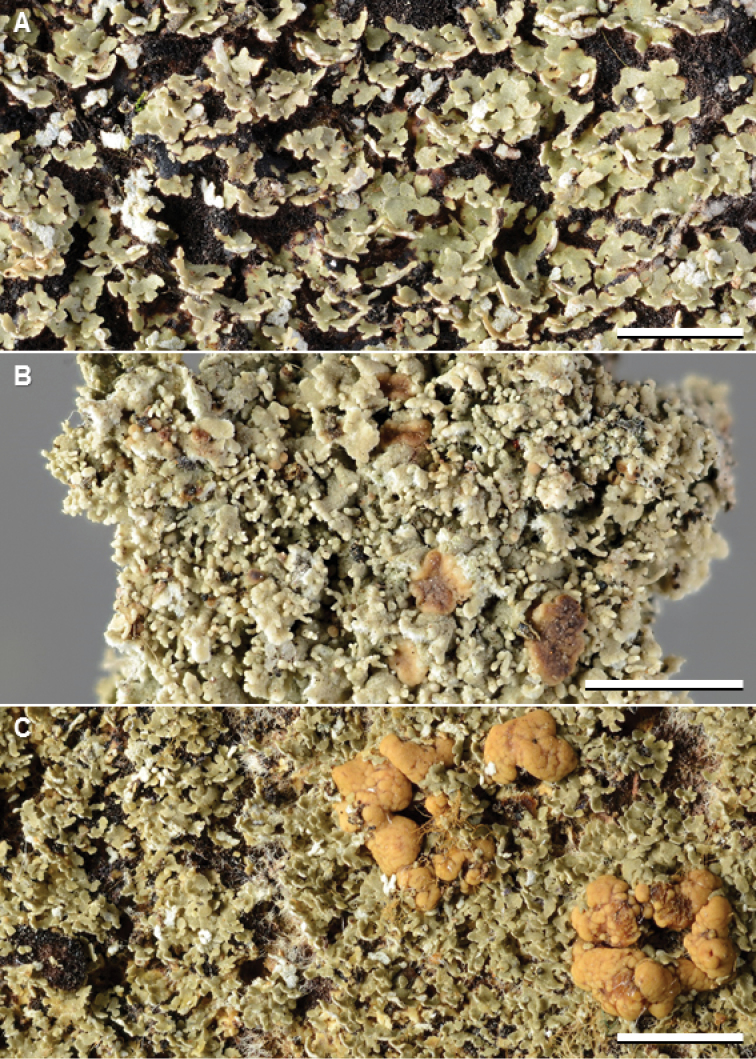
Species of *Phyllopsora* occurring in Asia and Melanesia. **A***Phyllopsoraporphyromelaena* (Kistenich & Weerakoon SK1-572) **B***P.pseudocorallina* sp. nov. (holotype, Kashiwadani 47806) **C***P.sabahana* sp. nov. (holotype, Wolseley, Thüs & Vairappan S.B.oQ.3). Scale bars: 2 mm.

**Figure 10.**
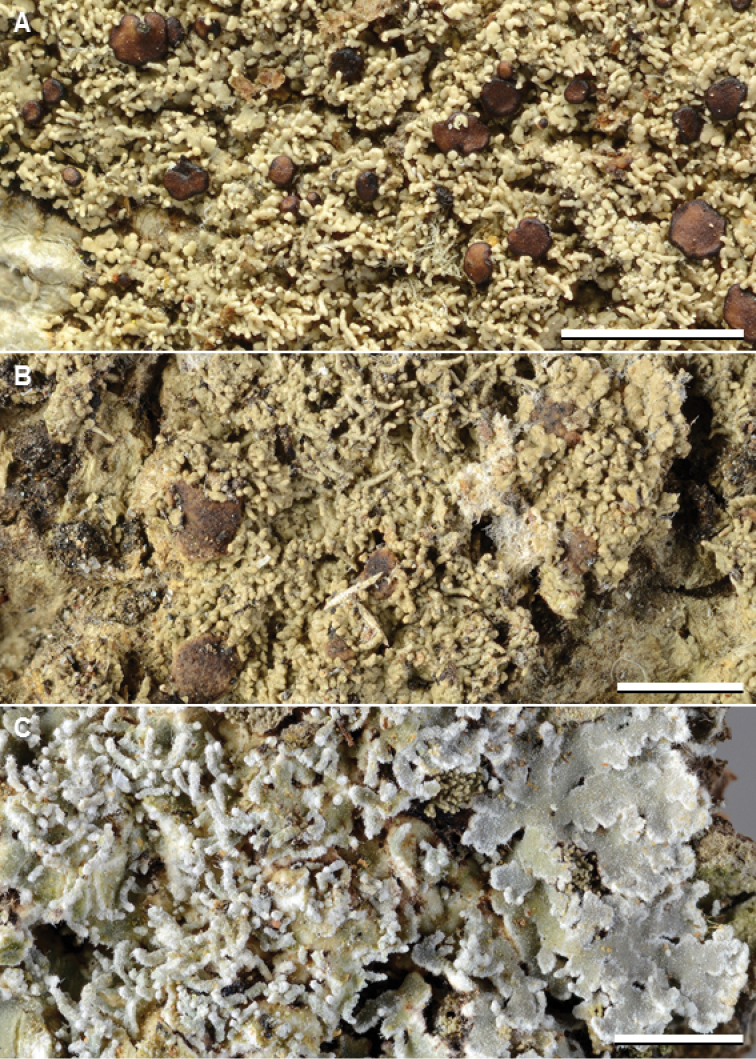
Species of *Phyllopsora* occurring in Asia and Melanesia. **A***Phyllopsorasantensis* (Aguirre, James & Wolseley 2485) **B***P.siamenses* sp. nov. (holotype, Wolseley & Boonpragob 3245) **C***P.subhispidula* (Weerakoon 1248). Scale bars: 2 mm.

In this study, we present the first revision of the genus *Phyllopsora* for Asia and Melanesia based on the integrative study of morphology, chemistry and DNA sequence data. We investigated 625 specimens of *Phyllopsora* collected from 18 countries and found the material to comprise at least 28 species of *Phyllopsora* s. str. (Figs 2–10) including three supported clades that we describe as species new to science. With this study, the genus *Phyllopsora* comprises 57 species.

Several species seem to be rather widespread throughout Asia and Melanesia, for instance, *P.castaneocincta*, *P.confusa*, *P.isidiosa*, and *P.porphyromelaena* (Table 1, Suppl. material 2: Table S1). In contrast, specimens of, for example, *P.cuyabensis*, *P.mediocris* and *P.neofoliata*, are rarely collected and reported from few countries (Table 1, Suppl. material 2: Table S1). Thus, their distribution range requires further studies.

Among the 28 species of *Phyllopsora*, eight are reported as new for Asia and Melanesia (Table 1). One of these new species is *P.africana* (Fig. 2A). This species has recently been found to be morphologically and chemically heterogeneous, comprising three chemotypes (Kistenich et al. in press). In addition to the known isidiate morph, a lacinulate morph was detected among *P.africana* material by Kistenich et al. (in press). Moreover, they described two new chemotypes. The lacinulate morph occurred in specimens of chemotype 1 and 3, but has so far never been found in those of chemotype 2. Specimens of chemotype 2, however, were shown to be morphologically cryptic to the sister species *P.swinscowii* (Kistenich et al. in press). In this study, we added twelve specimens of *P.africana* to our phylogeny (mainly lacinulate specimens of chemotype 3), but are not able to disentangle the difficult nature of this species complex. While we found most specimens of *P.africana* to roughly group according to chemotype in the phylogenetic tree (Suppl. material 1: Fig. S1), one specimen of *P.africana* chemotype 1 (7224) was more closely related to *P.swinscowii* (Suppl. material 1: Fig. S1). This raises the question of whether the two species should be synonymized based on their morphological and chemical similarity in combination with the short branches in the phylogenetic tree (Suppl. material 1: Fig. S1). We refrain from synonymizing them here, awaiting more data.

The two species *P.cuyabensis* (Fig. 4B) and *P.longiuscula* (Fig. 7B) are reported as new for the Asian continent. Specimens of both species are morphologically congruent with their Neotropical representatives. In the phylogenetic tree (Suppl. material 1: Fig. S1), however, the respective Asian accessions sit on rather long branches, clearly distinct from the Neotropical specimens. In these cases, there seem to exist genetically different populations for Neotropical and Asian specimens and more specimens should be collected to investigate the extent of genetic variation.

The genus *Phyllopsora* was recently shown to be polyphyletic by Kistenich et al. (2018b). The typical growth form, which characterizes this genus, has evolved multiple times independently in the family Ramalinaceae. These findings corroborate the morphological co-evolution in tropical lichens already indicated by Lakatos et al. (2006). Hence, molecular methods are often the only means of reliably assigning specimens to *Phyllopsora* or rather to its morphologically similar relatives (e.g., *Bacidia* De Not., *Bacidina* Vězda, *Eschatogonia*, *Parallopsora*). It is thus not surprising that several of our sequenced specimens (Table 3) were extraneous to *Phyllopsora* s. str. We did not assign those specimens to genus level, but all but one belong in the Ramalinaceae. The non-Ramalinaceae specimen appears to belong in the Malmideaceae. This indicates that correct taxonomic assignment even at family level using morphology may prove challenging in certain cases. Furthermore, about a quarter of the total material investigated could not be identified, partly because many of those unidentified specimens were sterile and deficient in lichen substances. Unfortunately, we were not able to generate sequences of all unidentified specimens in the course of this study.

### New species

The three new species, *P.pseudocorallina*, *P.sabahana* and *P.siamensis*, fall into distinct and well-supported clades in the phylogeny (Fig. 1A–C). They were originally assumed to comprise Asian populations of the species *P.porphyromelaena*, *P.corallina* and *P.imshaugii*, respectively, based on morphology and/or chemistry. Their sequence data, however, revealed them as separate species clearly distinct from their look-alikes (Fig. 1). *Phyllopsorapseudocorallina* (Fig. 9B) is distinguished from its namesake, i.e. *P.corallina*, by forming a partly more rosulate thallus. Poorly developed specimens, however, might be difficult to assign to the correct species. Specimens of *P.sabahana* (Fig. 9C) are challenging to identify based on morphology only. The species is morphologically and chemically almost identical to *P.porphyromelaena* chemotype 1. It differs only in forming slightly smaller ascospores. Thus, sterile specimens cannot be identified without DNA sequence data. *Phyllopsorasiamensis* (Fig. 10B) is described from material collected in Thailand and we have not been able to detect this species in collections from other countries. The specimens resemble *P.imshaugii* in morphology and chemistry, but may be readily distinguished by forming larger ascospores. See also the remarks in the Taxonomy section.

In addition, we found sequences of the four unidentified specimens with extraction numbers 1017, 7227, 7230, and 7231 to be resolved on rather long branches (Fig. 1, Suppl. material 1: Fig. S1). Hence, we could not assign them to any other *Phyllopsora* species, for which DNA sequences of the mtSSU or ITS region were available, based on molecular data, either. It is possible that these specimens represent several new species. In this study, however, we refrain from describing them as new species pending the collection of more material. Even though specimens 1017 and 7230 are clustered together in a clade with short branches (Suppl. material 1: Fig. S1), they are morphologically quite distinct and more specimens are needed to support the hypothesis that they belong to the same species.

### Unconfirmed species records

Despite investigating about 600 phyllopsoroid specimens, we were not able to find in our material any specimens belonging to seven species (i.e., *P.chlorophaea*, *P.corallina*, *P.isidiotyla*, *P.mauritiana*, *P.nemoralis*, *P.pyxinoides*, and *P.swinscowii*) previously reported from India, South Korea, Sri Lanka, Taiwan, Thailand, and Vietnam (Table 1), respectively. We have only investigated a few collections from especially India, South Korea, Taiwan, and Vietnam (Suppl. material 2: Table S1), though. Also for the other countries, collections are limited to certain areas and we cannot exclude the species’ occurrence in other parts of the respective countries. About 23% of the investigated material could not be identified to species level and it is possible that some of these unidentified specimens represent a poorly developed individual of any of these seven species. Regarding *P.corallina*, for instance, we found two candidate specimens from Papua New Guinea, but DNA sequence data is necessary to resolve their species status unambiguously. Alternatively, some of these species records might be based on misidentifications. In the case of *P.swinscowii*, we have shown the species to be morphologically identical to the isidiate morph of *P.africana*, a very widespread species. It is therefore possible that the records of *P.swinscowii* indeed represent *P.africana*. In general, we have repeatedly experienced difficulties in correctly identifying species of *Phyllopsora* based on morphology only. For many of the species records, it remains unclear whether anatomical studies and/or chemical investigations were performed as part of the identification process or not. Especially *P.chlorophaea*, *P.corallina* and *P.isidiotyla* may be difficult to identify without TLC or even sequence data.

## Taxonomy

This taxonomy section is a result of the integrative species delimitation process primarily based on the conclusions from the statistically inferred species delimitation analyses combined with morphological and chemical evaluations as performed in the global *Phyllopsora* study by Kistenich et al. (in press). The additional material of the present study complements the global dataset for the phylogenetic analysis (Fig. 1, Suppl. material 1: Fig. S1) and revealed three new species, which were mainly delimited by forming separate clades on long branches compared to their neighboring clades.

Distribution references for Asia and Melanesia are cited in Table 1; for all other distributions, references are cited below.

### Phyllopsora africana

Taxon classificationFungiLecanoralesRamalinaceae

Timdal & Krog

#### Description.

Timdal and Krog (2001), Elix (2009).

#### Distribution.

Africa (Timdal and Krog 2001), Asia, Australia (Elix 2009).

#### Remarks.

See discussion above and Kistenich et al. (in press) for taxonomic discussion. The species (Fig. 2A) is one of the most common in our material, represented by 59 collections (Suppl. material 2: Table S1). We found both isidate and lacinulate morphs as well as representatives of all three chemotypes (i.e., chemotype 1 contains chlorophyllopsorin and argopsin; chemotype 2 contains methyl 2,7-dichloropsoromate and methyl 2,7-dichloronorpsoromate; chemotype 3 contains chlorophyllopsorin, methyl 2,7-dichloropsoromate, methyl 2,7-dichloronorpsoromate, and argopsin) among the material. It is the phylogenetic sister to *P.swinscowii* (Fig. 1). The species is new to Asia and Melanesia, i.e. to Indonesia, Japan, Malaysia, Papua New Guinea, The Philippines, The Solomon Islands, Sri Lanka, Thailand, and Vanuatu.

### Phyllopsora breviuscula

Taxon classificationFungiLecanoralesRamalinaceae

(Nyl.) Müll. Arg.

#### Description.

Timdal and Krog (2001), Elix (2009).

#### Distribution.

Pantropical (Brako 1991, as P.parvifoliavar.breviuscula; Timdal and Krog 2001; Elix 2009).

#### Remarks.

Paleotropical material of this species (Fig. 2B) tends to be more narrow-lobed and ascending than Neotropical material. Species delimitation analyses by Kistenich et al. (in press) show that the taxon could be split into three or four entities, but as all sequenced specimens fell into one well-supported clade, and morphologically intermediate specimens exist, we still treat the taxon as one variable species. It is the phylogenetic sister to *P.mauritiana* (Fig. 1). The species is new to New Caledonia, The Philippines, and Vietnam.

### Phyllopsora buettneri

Taxon classificationFungiLecanoralesRamalinaceae

(Müll. Arg.) Zahlbr.

#### Description.

Swinscow and Krog (1981), Timdal and Krog (2001), Timdal (2008b, as *P.buettneri* chemotypes 1 and 2), Elix (2009).

#### Distribution.

Pantropical (Brako 1991, as P.buettnerivar.buettneri; Timdal and Krog 2001; Elix 2009).

#### Remarks.

We recognize five chemotypes of this species, three occurring in Asia and Melanesia (Fig. 2C). Chemotype 1 (pannarin and zeorin) was found in material from Japan, Sri Lanka, and Thailand; chemotype 3 (dechloropannarin, zeorin) in material from China, Japan, Sri Lanka, and Thailand; and chemotype 5 (pannarin and an unknown compound, no zeorin) in material from Papua New Guinea and Sri Lanka (Suppl. material 2: Table S1). The specimen of chemotype 1 from Japan, and one of the two from Sri Lanka, differed from typical specimens of the chemotype in lacking zeorin. Chemotype 5 is described here for the first time, and contains an unknown compound with R_f_ values similar to argopsin in solvent system B’ but with a distinct blue UV_366_ fluorescence (not quenching) on the chromatograms after development. Chemotype 2 (pannarin, phyllopsorin, zeorin) is Neotropical and chemotype 4 (argopsin, norargopsin, zeorin) is known from the Norfolk Islands. We were unable to sequence specimens of chemotype 4 and 5, but specimens of chemotype 1–3 are resolved in a clade with chemotype 1 and 2 of *P.porphyromelaena* (Fig. 1, Suppl. material 1: Fig. S1). The species is new to China, Japan, and Sri Lanka.

### Phyllopsora castaneocincta

Taxon classificationFungiLecanoralesRamalinaceae

(Hue) Kistenich & Timdal

#### Description.

Timdal and Krog (2001), Elix (2009), both as *P.kiiensis*.

#### Distribution.

Africa (Timdal and Krog 2001), Asia, Australia (Elix 2009).

#### Remarks.

This is one of the most common species in our material (Suppl. material 2: Table S1). It is usually easily recognized by the well-developed squamulose thallus on a reddish brown prothallus and by containing furfuraceic acid (Fig. 3A), but care is needed as about 10% of the examined specimens were actually deficient in lichen substances. It is new to Cambodia, Malaysia, Nepal, New Caledonia, Papua New Guinea, The Solomon Islands, Taiwan, and Thailand.

### Phyllopsora chodatinica

Taxon classificationFungiLecanoralesRamalinaceae

Elix

#### Description.

Elix (2006, 2009).

#### Distribution.

Australasia (Elix 2009) and Oceania.

#### Remarks.

This species resembles *P.porphyromelaena* and *P.sabahana*, to which it is closely related in the phylogenetic tree (Fig. 1), but differs in the presence of xanthones and the absence of argopsin and norargopsin. Kistenich et al. (in press) showed that probably all Neotropical records of this species (e.g., by Timdal 2008b, 2011) belong in another species, *P.neotinica*. *Phyllopsorachodatinica* (Fig. 3B) is new to Malaysia, New Caledonia, and Vanuatu.

### Phyllopsora cinchonarum

Taxon classificationFungiLecanoralesRamalinaceae

(Fée) Timdal

#### Description.

Brako (1989, as *Squamacidiajaneirensis*), Timdal (2008b), Elix (2009, as *Triclinumcinchonarum*).

#### Distribution.

Central and South America (Brako 1989; Timdal 2008b), Asia, Australia (Elix 2009).

#### Remarks.

The species is recognized by the squamulose thallus on a white prothallus, long isidia, and the presence of lobaric acid (Fig. 3C). Several additional compounds are reported, for example atranorin, fumarprotocetraric acid, and a scarlet pigment. In our Asian material, we have encountered only lobaric acid (always major), atranorin (minor to absent), and some unknown compounds (minor to absent). It is the phylogenetic sister to the Neotropical *P.concinna* (Fig. 1).

### Phyllopsora confusa

Taxon classificationFungiLecanoralesRamalinaceae

Swinscow & Krog

#### Description.

Swinscow and Krog (1981), Timdal and Krog (2001), Elix (2009).

#### Distribution.

Pantropical (Brako 1991; Timdal and Krog 2001; Elix 2009).

#### Remarks.

This species is characterized by the small, lacinulate squamules lacking lichen substances (Fig. 4A), but may be difficult to separate from, for example, *P.foliata* and *P.mediocris*. It is also possible that some of the specimens we have left undetermined belong in this species. We have sequenced nine specimens from Asia and Melanesia (Table 2), in addition to the holotype from Kenya, and those specimens make up the core in our concept of this species. The accessions of *P.confusa* form a strongly supported clade with *P.loekoesii* in the phylogenetic tree (Fig. 1). The two specimens from Papua New Guinea (6360 and 6361) fall in between the *P.confusa* and *P.loekoesii* clade (Suppl. material 1: Fig. S1) and show an intermediate morphology. Further specimens are needed to investigate the possible synonymy of these two species. *Phyllopsoraconfusa* is new to Indonesia, Japan, Malaysia, Taiwan, and Thailand.

### Phyllopsora cuyabensis

Taxon classificationFungiLecanoralesRamalinaceae

(Malme) Zahlbr.

#### Description.

Timdal (2008b).

#### Distribution.

Central and South America (Brako 1991; Timdal 2008b), Asia.

#### Remarks.

The species is represented by a single specimen from Thailand in our material (Fig. 4B). The Asian accession (450) falls into a strongly supported clade with sequences from four Neotropical specimens, although resolved on a long branch as sister to all Neotropical accessions (Suppl. material 1: Fig. S1). Being morphologically identical to the Neotropical specimens, it is unclear whether this specimen (450) represents a new species or merely genetic variation within *P.cuyabensis*. Additional sequences of Asian specimens are necessary to evaluate this possibility further. The species is sister to a clade comprising *P.byssiseda*, *P.fendleri* and *P.kalbii* (Fig. 1). The species is new to Asia.

### Phyllopsora dolichospora

Taxon classificationFungiLecanoralesRamalinaceae

Timdal & Krog

#### Description.

Timdal and Krog (2001).

#### Distribution.

Africa (Timdal and Krog 2001), Asia.

#### Remarks.

This species (Fig. 4C) is morphologically and chemically (furfuraceic acid) similar to *P.furfuracea*, to which it is closely related (Fig. 1), but differs in forming longer ascospores and in containing additional substances (methyl furfuraceiate and methyl homofurfuraceiate). Judging from the number of examined specimens (Suppl. material 2: Table S1), *P.dolichospora* seems to be more common than *P.furfuracea* in Asia, although the number of reports (Table 1) suggests the opposite. This, however, might be a result of morphological misidentifications when TLC has not been run. The species is new to Japan and Papua New Guinea.

### Phyllopsora foliata

Taxon classificationFungiLecanoralesRamalinaceae

(Stirt.) Zahlbr.

#### Description.

Elix (2009).

#### Distribution.

Asia, Australia (Elix 2009).

#### Remarks.

This rarely reported Australian species (Fig. 5A) is here confirmed from Japan and Sri Lanka mainly based on our DNA sequences (both mtSSU and ITS), which were compared with sequences obtained from Australian material (Table 2). It is new to Japan.

### Phyllopsora furfuracea

Taxon classificationFungiLecanoralesRamalinaceae

(Pers.) Zahlbr.

#### Description.

Timdal and Krog (2001), Timdal (2008b), Elix (2009).

#### Distribution.

Pantropical (Brako 1991; Timdal and Krog 2001; Elix 2009).

#### Remarks.

Despite widespread reports in the literature, we were able to confirm the presence of this species (Fig. 5B) in Papua New Guinea, Sri Lanka, and Thailand, only. In the phylogenetic tree, *P.furfuracea* forms a clade with *P.dolichospora* and *P.foliatella* (Fig. 1).

### Phyllopsora gossypina

Taxon classificationFungiLecanoralesRamalinaceae

(Sw.) Kistenich, Timdal, Bendiksby & S. Ekman

#### Description.

Hue (1909).

#### Distribution.

Apparently pantropical.

#### Remarks.

The species (Fig. 5C) was included in the genus *Crocynia* until recently (Kistenich et al. 2018b), and not originally a part of our taxon sampling; hence the few specimens examined. The accession from Sri Lanka (7201) clusters together with specimens of *C.molliuscula* (Suppl. material 1: Fig. S1), from which it is morphologically and chemically different. Further specimens need to be investigated to inform about its relationship to *C.molliuscula*. The species is the phylogenetic sister to *P.imshaugii* (Fig. 1).

### Phyllopsora halei

Taxon classificationFungiLecanoralesRamalinaceae

(Tuck.) Zahlbr.

#### Description.

Swinscow and Krog (1981, as *P.pannosa*), Timdal and Krog (2001).

#### Distribution.

North America (Brako 1991), Africa (Timdal and Krog 2001), Asia.

#### Remarks.

This species (Fig. 6A) was previously known from the type collection from North America (Louisiana), East Africa (Ethiopia, Kenya, Tanzania), and a few reports from Asia (Table 1). We here confirm its presence in Asia, based on DNA sequences from material from Sri Lanka compared with sequences from Kenya and Tanzania (Suppl. material 1: Fig. S1). Three chemotypes of this species are known (Timdal and Krog 2001), differing in terpenoid patterns and presence of an unknown compound. Our two specimens from Sri Lanka belong in chemotype 3 of Timdal and Krog (2001). The species is the phylogenetic sister to *P.amazonica* (Fig. 1). It is new to Sri Lanka.

### Phyllopsora himalayensis

Taxon classificationFungiLecanoralesRamalinaceae

G.K. Mishra, Upreti & Nayaka

#### Description.

Mishra et al. (2011).

#### Distribution.

India (Mishra et al. 2011).

#### Remarks.

The species was not studied by us due to lack of response from LWG to our repeated loan requests.

### Phyllopsora isidiosa

Taxon classificationFungiLecanoralesRamalinaceae

Kistenich & Timdal

#### Description.

Kistenich et al. (in press).

#### Distribution.

Pantropical, also occurring in temperate Asia and North America (Kistenich et al. in press).

#### Remarks.

The species (Fig. 6B) is treated in detail by Kistenich et al. (in press). It is closely related to, for instance, *P.dolichospora*, *P.foliatella*, and *P.furfuracea* (Fig. 1).

### Phyllopsora kalbii

Taxon classificationFungiLecanoralesRamalinaceae

Brako

#### Description.

Brako (1991), Timdal and Krog (2001).

#### Distribution.

North, Central, and South America (Brako 1991), Africa (Timdal and Krog 2001), Asia.

#### Remarks.

The species (Fig. 6C) was reported from India by Mishra et al (2011), and we confirm its presence in Asia by DNA sequences (mtSSU and ITS; 456) from material from Thailand. The species is sister to a clade comprising *P.byssiseda* and *P.fendleri* (Fig. 1). It is new to Thailand.

### Phyllopsora loekoesii

Taxon classificationFungiLecanoralesRamalinaceae

S.Y. Kondr., E. Farkas, S.–O. Oh & Hur

#### Description.

Kondratyuk et al. (2016).

#### Distribution.

Asia.

#### Remarks.

The species (Fig. 7A) was recently described from South Korea by Kondratyuk et al. (2016), and we report it as new to Japan and Nepal. Our sequences were compared to unpublished sequences of the holo- and isotype kindly provided to us by Sergey Kondratyuk. Our accessions form a strongly supported clade together with accessions of *P.confusa* (Fig. 1), from which it is difficult to distinguish. See also remarks for *P.confusa*.

### Phyllopsora longiuscula

Taxon classificationFungiLecanoralesRamalinaceae

(Nyl.) Zahlbr.

#### Description.

Brako (1991).

#### Distribution.

Central and South America (Brako 1991), Asia, Australia (Kistenich et al. in press).

#### Remarks.

In the concept of Brako (1991) and Timdal (2011), this species is lacinulate. Kistenich et al. (in press), however, extend the concept to include the isidiate species *P.intermediella*, which they synonymize. The Asian material we have examined is lacinulate. The species (Fig. 7B) is the phylogenetic sister to *P.thaleriza* (Fig. 1). It is new to Asia (Sri Lanka, Thailand, and Vietnam).

### Phyllopsora mediocris

Taxon classificationFungiLecanoralesRamalinaceae

Swinscow & Krog

#### Description.

Swinscow and Krog (1981), Timdal and Krog (2001).

#### Distribution.

Africa (Timdal and Krog 2001), Asia.

#### Remarks.

The species (Fig. 7C) was previously known from East Africa and the Mascarenes (Timdal and Krog 2001). Although not sequenced, we here report it as new to Asia based on a specimen (Moberg 2750, UPS) from Sri Lanka (Suppl. material 2: Table S1). The species is the phylogenetic sister to *P.parvifolia* (Fig. 1).

### Phyllopsora neofoliata

Taxon classificationFungiLecanoralesRamalinaceae

Elix

#### Description.

Elix (2006, 2009).

#### Distribution.

Africa (Kistenich et al. in press), Asia, Australia (Elix 2009).

#### Remarks.

This originally Australian species is reported as new to Africa (Kenya) by Kistenich et al. (in press) and here as new to Asia (Sri Lanka; Fig. 8A). Both the African and Sri Lankan specimens were sequenced (mtSSU and ITS) and found to conform with sequences of an isotype (O L-1319). The species is generally identified by the squamulose, lacinulate thallus containing furfuraceic acid.

### Phyllopsora parvifolia

Taxon classificationFungiLecanoralesRamalinaceae

(Pers.) Müll. Arg.

#### Description.

Elix (2009).

#### Distribution.

Pantropical, but mainly Neotropical, extending into the temperate zones in North and South America and in Europe (Brako 1991, as P.parvifoliavar.parvifolia; Kistenich et al. in press).

#### Remarks.

Despite several reports from Asia and Melanesia (Table 1), we have seen only a single specimen of this species (Sri Lanka, Kistenich & Weerakoon SK1-661, PDA, not sequenced; Fig. 8B) from the area. The species is the phylogenetic sister to *P.mediocris* (Fig. 1). It is new to Sri Lanka.

### Phyllopsora parvifoliella

Taxon classificationFungiLecanoralesRamalinaceae

(Nyl.) Müll. Arg.

#### Description.

Timdal (2008b).

#### Distribution.

Central and South America (Brako 1991; Timdal 2008b), Asia.

#### Remarks.

This squamulose, isidiate species contains atranorin and parvifoliellin (Fig. 8C); characters it shares with *P.concinna* and *P.rappiana*. The molecular phylogeny (Fig. 1) shows that the three species are not closely related, though; rather *P.parvifoliella* belongs in a clade together with *P.africana*, *P.ochroxantha*, and *P.swinscowii*. We have sequenced material from Indonesia and Thailand, and here report the species as new to Asia and Melanesia, i.e. from Indonesia, Papua New Guinea, The Philippines, and Thailand.

### Phyllopsora porphyromelaena

Taxon classificationFungiLecanoralesRamalinaceae

(Vain.) Zahlbr.

#### Description.

Timdal and Krog (2001), Elix (2009), both as *P.albicans*.

#### Distribution.

Pantropical (Brako 1991, as P.buettnerivar.glauca chemical stains I and III; Timdal and Krog 2001; Elix 2009).

#### Remarks.

This is the most common species in our material from Asia and Melanesia (Fig. 9A), despite previous records only from India, The Philippines, and Taiwan (mostly as *P.albicans*). Two chemotypes are previously recognized, and a third is recognized here. Chemotype 1 (argopsin and norargopsin) and chemotype 2 (argopsin and pannarin) are both widely distributed in Asia and Melanesia, but chemotype 3 (zeorin and three unknown compounds) is restricted to Thailand (Suppl. material 2: Table S1). The unknown compounds move in R_f_-classes A:3–4, B’:4–5, C:5 (major compound); A:6, B’:6, C:5–6 (minor compound); and A:3, B’:3, C:5 (minor compound).

In the phylogenetic tree (Fig. 1, Suppl. material 1: Fig. S1), accessions of chemotypes 1 and 2 group into a weakly supported clade with *P.buettneri*, while accessions of chemotype 3 form a clade with *P.chodatinica* and the *P.buettneri*/*P.porphyromelaena* clade. Additional specimens of chemotype 3 should be sequenced to find out whether it indeed represents a chemical strain of *P.porphyromelaena* or rather a distinct species.

The species is morphologically very similar to *P.sabahana*; see that species for discussion. It is possible that some specimens listed as *P.porphyromelaena* chemotype 1 in Suppl. material 2: Table S1, especially those from Malaysia, represent *P.sabahana*. It is new to Fiji, Indonesia, Japan, Malaysia, New Caledonia, Papua New Guinea, South Korea, Sri Lanka, and Thailand.

### Phyllopsora pseudocorallina

Taxon classificationFungiLecanoralesRamalinaceae

Kistenich & Timdal sp. nov

829572

Fig. 9B

#### Diagnosis.

Differs from *P.corallina* in having a more rosulate thallus and in substitutions in the mtSSU and ITS sequences.

#### Type.

CAMBODIA, *Siem Reap*: around Ta Nei temple, Angkor Wats complex, 13°27'N, 103°53'E, ca. 30 m alt., on rock (sand stone), 2005-12-20, H. Kashiwadani 47806 (TNS!—holotype) [TLC: no lichen substances; DNA: MK412430 (mtSSU), MK412491 (ITS)].

#### Description.

Thallus effuse or forming irregular rosettes up to 1 cm diam., squamulose; squamules medium sized, up to 1 mm wide, adnate to ascending, elongate, contiguous or partly imbricate, crenulate to incised, plane to weakly convex, medium green, glabrous on the upper side, faintly pubescent along the margin; isidia common, attached marginally to the squamules, cylindrical, simple or slightly branched, up to 0.1 mm wide and 0.6 mm long; upper cortex formed by thick-walled hyphae with rounded lumina (type 2), 20–30 µm thick; cortex and medulla not containing crystals (PD–, K–); prothallus indistinct to partly well developed, white.

Apothecia common, up to 1.5 mm diam., rounded when young, later often becoming irregular, simple or sometimes somewhat conglomerate, plane to moderately convex, yellowish to medium brown, with an indistinct, usually slightly paler, glabrous to finely pubescent margin; excipulum pale brown to colourless, K–; hypothecium pale brown to colourless, K–; epithecium colourless; no crystals in apothecium; ascospores narrowly ellipsoid to shortly bacilliform, simple, 6–10 × 2.5–3 µm (n=30). Conidiomata not seen.

#### Chemistry.

No lichen substances.

#### Distribution.

Cambodia, Malaysia, Papua New Guinea, The Seychelles.

#### Etymology.

The specific epithet refers to its morphological and chemical similarity to *P.corallina*.

#### Remarks.

The species is morphologically, anatomically, and chemically very similar to *P.corallina*. There is, however, a tendency of *P.pseudocorallina* being more rosulate, i.e., composed of more radiating and elongated marginal lobes. The phylogenetic tree (Fig. 1), however, shows the two species not to be closely related: *P.corallina* is resolved in a clade with *P.glaucella* and *P.rappiana* as sister to *P.martinii*, while *P.pseudocorallina* appears in a clade with *P.castaneocincta* and *P.neofoliata* among others (Fig. 1). The new species is widely distributed in Asia and also found on The Seychelles. It is unclear whether *P.corallina* occurs in Asia at all or if previously reported specimens of *P.corallina* rather represent specimens of *P.pseudocorallina*.

#### Additional specimens examined.

MALAYSIA, *Sabah*: Malaysian Borneo, Maliau, “Knowledge Trail”, pristine lowland dipterocarp forest; on stem (ca. 20 m high) of fallen tree on crushed bridge, 2012, H. Thüs, P. Wolseley & C. Vairappan M001a (BORH) [DNA: MK412437 (mtSSU)]; same locality data, H. Thüs, P. Wolseley & C. Vairappan M001b (BORH); same locality data, H. Thüs, P. Wolseley & C. Vairappan M005 (BORH) [DNA: MK412438 (mtSSU)]. PAPUA NEW GUINEA, *Madang*: Manam island, near Bogia, in gardens near Budua, 4°07'S, 145°00'E, 50 m alt., epiphytic, 1992-07-22, P. Diederich 11386 (hb. Diederich) [DNA: MK412449 (mtSSU), MK412495 (ITS)]. THE SEYCHELLES, *Mahé*: W of Anse Royale, Le Jardin du Roi, 4.74642S, 55.50297E, 150–200 m alt., parkland and neighbouring forest, on rock, 2015-07-26, P. Diederich 17809 (hb. Diederich); Port Glaud, near Sauzier Waterfall, 4.65847S, 55.41403E, 20–70 m alt., on tree, 2015-07-28, P. Diederich 17897 (hb. Diederich).

### Phyllopsora sabahana

Taxon classificationFungiLecanoralesRamalinaceae

Kistenich & Timdal sp. nov.

829571

Fig. 9C

#### Diagnosis.

Differs from *P.porphyromelaena* in having smaller ascospores and in substitutions in the mtSSU and ITS sequences.

#### Type.

MALAYSIA, *Sabah*: Malaysian Borneo, SAFE-project Area, mostly Macaranga dominated secondary forest, 2012, P. Wolseley, H. Thüs & C. Vairappan S.B.oQ.3 (BORH!—holotype) [TLC: argopsin (major), norargopsin (minor); DNA: MK412434 (mtSSU)].

#### Description.

Thallus effuse, squamulose; squamules medium sized, up to 0.8 mm wide, ascending, elongated, often imbricate, incised to deeply divided, plane to weakly convex; upper side pale green to medium green, glabrous, epruinose; margin concolorous with upper side, often finely pubescent; lacinules common, developing from lobe-tips; upper cortex formed by thick-walled hyphae with cylindrical lumina (type 1), 30–40 µm thick, containing crystals dissolving in K (PD+ orange, K–); medulla containing crystals partly dissolving in K (PD+ orange, K–); prothallus well developed, reddish brown.

Apothecia not common, up to 2 mm diam., rounded to irregular, simple or soon becoming conglomerate, weakly to moderately convex, yellowish brown, more or less immarginate even when young; excipulum pale brown to colourless, K–; hypothecium medium brown, K–; epithecium pale brown to colorless; no crystals in apothecia; ascospores narrowly ellipsoid, simple, 6–8 × 2–2.5 µm (n=20). Conidiomata not seen.

#### Chemistry.

Argopsin (major), norargopsin (minor). Medulla and upper cortex PD+ orange, K–, C–, KC–.

#### Distribution.

Malaysia (Borneo).

#### Etymology.

The specific epithet refers to its occurrence in Sabah, Malaysia.

#### Remarks.

The species is morphologically and chemically very similar to *P.porphyromelaena* chemotype 1, and is close to be regarded as a morphologically cryptic species. It may, however, be distinguished in forming smaller ascospores (6–8 × 2–2.5 vs. 8–13 × 2–4 µm). Apothecia are not common in neither species, however, and the measurements are based on only 20 spores from each species (the holotype of *P.sabahana* and two specimens of *P.porphyromelaena* from La Réunion). In the phylogenetic tree (Fig. 1), the five accessions of *P.sabahana* form a strongly supported clade as sister to the Neotropical species *P.neotinica*, from which it may readily be distinguished in its composition of lichen substances (*P.neotinica* contains xanthones). So far, *P.sabahana* is only known from Borneo.

#### Additional specimens examined.

MALAYSIA, *Sabah*: Malaysian Borneo: Maliau Basin, surroundings of Agathis Camp, pristine lowland Dipterocarp forest, 2012, P. Wolseley, H. Thüs & C. Vairappan, C. M089 (BORH) [DNA: MK412441 (mtSSU)]; Danum valley, pristine lowland Dipterocarp forest, 2012, P. Wolseley, H. Thüs & C Vairappan D.8.02.4 (BORH) [DNA: MK412442 (mtSSU)]; Ranau district, Kinabalu park, Tambuyukon trail, Kera camp (loc. T089), 6°12.742'N, 116°43.609'E, 728 m alt., epiphytic, 2014-12-08, A. Paukov 2229 (B) [DNA: MK412455 (mtSSU), MK412498 (ITS)] & 2230 (B) [DNA: MK412453 (mtSSU), MK412496 (ITS)].

### Phyllopsora santensis

Taxon classificationFungiLecanoralesRamalinaceae

(Tuck.) Swinscow & Krog

#### Description.

Timdal (2008b), Elix (2009).

#### Distribution.

North, Central, and South America (Brako 1991, as P.corallinavar.santensis; Timdal 2008b), Asia, Australia (Elix 2009).

#### Remarks.

The species was previously reported from Japan, Papua New Guinea, and The Philippines (Table 1), and is here reported from four localities in Thailand (Fig. 10A). We were unable to produce DNA sequences from our material, and the identification is based on typical morphology and presence of argopsin (major) and noragopsin (minor). New to Thailand.

### Phyllopsora siamensis

Taxon classificationFungiLecanoralesRamalinaceae

Kistenich & Timdal sp. nov.

829573

Fig. 10B

#### Diagnosis.

Differs from *P.imshaugii* in having more well developed squamules, larger ascospores, and in substitutions in the mtSSU and ITS sequences.

#### Type.

THAILAND, *Lampang*: Doi Khun Tan National Park, loc. T118, 18°25'N, 99°14'E, 1000 m alt., hill evergreen forest, 1993-01-11, P.A. Wolseley & K. Boonpragob 3245 (BM 749856!—holotype) [TLC: norstictic acid; DNA MK412410 (mtSSU), MK412477 (ITS)].

#### Description.

Thallus effuse, crustose to squamulose; squamules small, up to 0.4 mm wide, adnate, isodiametrical, more or less scattered when young, later contiguous or fusing, more or less crenulate, plane to weakly convex; upper side medium green, somewhat shiny, epruinose, glabrous; margin concolorous with upper side, often pubescent; isidia common, attached marginally to the squamules, cylindrical, simple or slightly branched, up to 0.15 mm wide and 1.5 mm long; upper cortex formed by thick-walled hyphae with rounded lumina (type 2), 15–30 µm thick, containing a few scattered crystals dissolving in K; medulla containing crystals dissolving in K and recrystallizing by forming acicular, red crystals, PD+ yellow, K+ red; prothallus well developed, thick, white.

Apothecia seen in the holotype only, up to 1.5 mm diam., more or less plane when young, soon becoming weakly to moderately convex, medium brown, rounded to irregular, simple, when young with a rather thick, paler, weakly pubescent margin, later becoming more or less immarginate; excipulum pale brown in the rim, darker brown in inner part; hypothecium dark brown, K–; crystals present in inner part of exciple and in hypothecium, dissolving in K and recrystallizing by forming acicular, red crystals; epithecium pale brown to colourless, K–; ascospores narrowly ellipsoid or fusiform to bacilliform, simple, 15–22 × 3.5–4.5 µm (n=20). Conidiomata not seen.

#### Chemistry.

Norstictic acid (major), atranorin (minor to trace or absent). Medulla PD+ yellow, K+ red, C–, KC–.

#### Distribution.

Thailand.

#### Etymology.

The specific epithet refers to its occurrence in Thailand.

#### Remarks.

The species is morphologically and chemically very similar to *P.imshaugii*. *Phyllopsorasiamensis*, however, may be distinguished by forming slightly larger squamules and longer ascospores (15–22 × 3.5–4.5 vs 10.5–14.5 × 3–4 µm; the latter measurements are based on 40 spores in the type material from Jamaica) than *P.imshaugii*. So far, *P.imshaugii* is only known to occur in the Neotropics, while *P.siamensis* is solely known from Thailand. In the phylogenetic tree (Fig. 1), the four accessions of *P.siamensis* cluster in a strongly supported clade as sister to a clade comprising *P.cinchonarum* and *P.concinna*, from which the new species is readily distinguished by its chemistry. *Phyllopsoraimshaugii* and *P.siamensis* are the only *Phyllopsora* species known to contain norstictic acid; the major compound of the two other species are lobaric acid and parvifoliellin, respectively.

#### Additional specimens examined.

THAILAND, *Chiang Mai*: Doi Suthep National Park headquarters walk, loc. 62.4, 18°48'N, 98°54'E, 1050 m alt., tropical mixed deciduous forest, on Lauraceae, 1993-03-27, B. Aguirre–Hudson & P.A. Wolseley 1643 (BM 749866) [DNA: MK412419 (mtSSU), MK412485 (ITS)]; *Uthai Thani*: Khao Nang Rum, Cathouse site, 15°29'N, 99°18'E, 650 m alt., tropical mixed deciduous forest, 1992-01-07, P.A. Wolseley & B. Aguirre–Hudson 5580 p.p. (BM 1031552 p.p.) [DNA: MK412423 (mtSSU), MK412487 (ITS)]; Khao Nang Rum, Khao Kiew, 15°27'N, 99°20'E, 1250 m alt., oak/chestnut forest, 1992-01-23, P.A. Wolseley and Onsar 5590 (BM 749833) [DNA: MK412418 (mtSSU), MK412484 (ITS)].

### Phyllopsora subhispidula

Taxon classificationFungiLecanoralesRamalinaceae

(Nyl.) Kalb & Elix

#### Description.

Timdal and Krog (2001).

#### Distribution.

Africa (Timdal and Krog 2001), Asia.

#### Remarks.

This species resembles closely the more common *P.buettneri*, but differs in forming isidia, not lacinules (Fig. 10C). It contains argopsin (major), norargopsin (minor), zeorin (major), and sometimes atranorin (trace), similar to chemotype 4 of *P.buettneri*. Phylogenetically (Fig. 1), the two species are not closely related, though. *Phyllopsorasubhispidula* is sister to the clade comprising *P.nemoralis* and *P.hispaniolae*/*P.rosei* (Fig. 1). It is new to Asia (Sri Lanka).

### Key to the phyllopsoroid genera in Asia and Melanesia

**Table:** 

1	Apothecia zeorine, surrounded by a thalline sheath	***Physcidia* p.p.**
–	Apothecia biatorin	**2**
2(1)	Tholus non-amyloid or with an indistinct conical amyloid structure; ascospores filiform, spirally arranged in ascus; thallus and apothecia with red or purple patches caused by non-crystalline, acetone-insoluble pigment	*** Krogia ***
–	Tholus with a distinct amyloid conical structure (*Bacidia* type); ascospores ellipsoid to filiform, not spirally arranged in ascus; thallus and apothecia without red patches	**3**
3(2)	Upper and lower cortices formed by a single layer of isodiametric cells, continuous over the edge of the areolae/squamule	*** Eschatogonia ***
–	Upper cortex multicellular or poorly differentiated; lower cortex absent	**4**
4(2)	Ascospores ellipsoid to fusiform, simple or rarely pseudoseptate	**5**
–	Ascospores bacilliform to filiform, septate or pseudoseptate	**6**
5(4)	Apothecia and prothallus blackish brown to black; isidia lacking; thallus containing fumarprotocetraric acid	‘***Phyllopsora*’ cfr. *nigrocincta* (Malmideaceae)**
–	Apothecia brown; prothallus white to dark reddish brown; isidia present or absent; if fumarprotocetraric acid present, then isidia present	*** Phyllopsora ***
6(4)	Thallus sorediat	**7**
–	Thallus not sorediate	**8**
7(6)	Squamules mostly adnate, bursting into convex soralia, containing atranorin and divaricatic acid	‘***Phyllopsora*’ *sorediata***
–	Squamules ascending, with labriform soralia, containing methyl barbatate and often terpenoids	‘***Phyllopsora*’ *glaucescens***
8(6)	Thallus large, subfoliose, isidiate	‘***Physcidia*’ *cylindrophora***
–	Thallus crustose to squamulose, not isidiate	**9**
9(8)	Thallus formed by ascending squamules, lacinulate, containing stictic acid	*Parallopsora* sp.^*^
–	Thallus crustose or formed by adnate squamules, not lacinulate, not containing lichen substances	**10**
10(9)	Thallus crustose	*** Sporacestra ***
–	Thallus squamulose	*** Aciculopsora ***

### Key to the species of *Phyllopsora* in Asia and Melanesia

**Table:** 

1	Thallus pruinose, rosulate, broad-lobed	**2**
–	Thallus not pruinose, effuse to rosulate, narrow to broad-lobed	**3**
2(1)	Thallus lacinulate, containing pannarin, dechloropannarin or rarely argopsin	*** P. buettneri ***
–	Thallus isidiate, containing argopsin	*** P. subhispidula ***
3(1)	Upper cortex absent or poorly developed	**4**
–	Upper cortex well developed	**5**
4(3)	Species always apotheciate; isidia lacking; apothecia plain to concave, with a pale margin; lichen substances present	*** P. gossypina ***
–	Species apotheciate or not; isidia often present; apothecia convex, more or less immarginate; lichen substances absent	*** P. cuyabensis ***
5(3)	Medulla K+ red (norstictic acid)	*** P. siamensis ***
–	Medulla K–	**6**
6(5)	Medulla PD+ orange to red	**7**
–	Medulla PD–	**10**
7(6)	Prothallus white or absent; lacinules absent	*** P. santensis ***
–	Prothallus brown; lacinules present or absent	**8**
8(7)	Squamules isodiametrical or shortly elongate, more or less adnate, containing chlorophyllopsorin or methyl 2,7-dichloronorpsoromate; isidia or lacinules present	*** P. africana ***
–	Squamules elongate, ascending, lacking chlorophyllopsorin and methyl 2,7-dichloronorpsoromate; lacinules present	**9**
9(8)	Ascospores narrowly ellipsoid, 6–8 × 2–2.5 µm	*** P. sabahana ***
–	Ascospores narrowly ellipsoid to fusiform or bacilliform, 8.0–12.5 × 2.5–3.5 µm	*** P. porphyromelaena ***
10(6)	Thallus isidiate	**11**
–	Thallus phyllidiate, lacinulate or apotheciate	**20**
11(10)	Prothallus white or absent	**12**
–	Prothallus brown	**15**
12(11)	Isidia often more than 1 mm long, mainly simple; ascospores bacilliform to acicular, 26–41 × 2–3 µm; containing lobaric acid	*** P. cinchonarum ***
–	Isidia shorter than 1 mm, globular to coralloid; ascospores ellipsoid to shortly bacilliform, less than 12 µm long; containing only atranorin or no lichen substance	**13**
13(12)	Isidia globular; thallus containing atranorin; crystals present in medulla and hypothecium	*** P. himalayensis ***
–	Isidia cylindrical to coralloid; thallus and apothecia lacking lichen substances and crystals	**14**
14(13)	Isidia becoming coralloid; species crustose, effuse; areoles up to 0.1 mm diameter	*** P. isidiosa ***
–	Isidia cylindrical or weakly branched; species squamulose, effuse or rosulate; squamules up to 1 mm diameter	*** P. pseudocorallina ***
15(11)	Thallus crustose, consisting of more or less scattered areoles or sometimes isidia only	**16**
–	Thallus squamulose	**17**
16(15)	Ascospores narrowly ellipsoid, 7–13 × 2–3 µm; containing furfuraceic acid only	*** P. furfuracea ***
–	Ascospores bacilliform, 16–25 × 2–3 µm; containing furfuraceic acid and 2–3 related compounds	*** P. dolichospora ***
17(15)	Prothallus thick, forming a cushion with colonizing areoles along the periphery	**18**
–	Prothallus thin, not forming a cushion	**19**
18(17)	Thallus containing atranorin and terpenoids	*** P. halei ***
–	Thallus containing furfuraceic acid or rarely no compounds	*** P. castaneocincta ***
19(17)	Isidia globular or shortly cylindrical; thallus pale green, containing atranorin or no lichen substances	*** P. kalbii ***
–	Isidia cylindrical; thallus dark green to brown, containing atranorin and parvifoliellin	*** P. parvifoliella ***
20(10)	Thallus containing xanthones	*** P. chodatinica ***
–	Thallus not containing xanthones	**21**
21(20)	Thallus containing furfuraceic acid	*** P. neofoliata ***
–	Thallus not containing lichen substances	**22**
22(21)	Prothallus brown, well developed	**23**
–	Prothallus white or absent	**27**
23(22)	Thallus rosulate or composed of elongated squamules	**24**
–	Thallus effuse, composed of more or less isodiametrical squamules	**26**
24(23)	Thallus phyllidiate; phyllidia mainly occurring in central part of thallus	*** P. parvifolia ***
–	Thallus lacinulate	**25**
25(24)	Squamules long, linear, deeply incised to branched	*** P. breviuscula ***
–	Squamules short, crenulate to narrowly incised	*** P. mediocris ***
26(23)	Thallus crustose, consisting of closely adnate areoles and ascending lacinules	*** P. longiuscula ***
–	Thallus squamulose, consisting of ascending squamules, breaking into lacinules	*** P. confusa ***
27(22)	Thallus phyllidiate; phyllidia mainly occurring in central part of thallus	*** P. parvifolia ***
–	Thallus lacinulate	**28**
28(27)	Squamules closely adnate, elongated, linear, somewhat branched	*** P. loekoesii ***
–	Squamules ascending, short, not branched	**29**
29(28)	Ascospores narrowly ellipsoid to fusiform, 11–20 × 2–3 µm	*** P. foliata ***
–	Ascospores narrowly ellipsoid to shortly bacilliform, 9–11 × 2–2.5 µm	*** P. confusa ***

## Supplementary Material

XML Treatment for Phyllopsora africana

XML Treatment for Phyllopsora breviuscula

XML Treatment for Phyllopsora buettneri

XML Treatment for Phyllopsora castaneocincta

XML Treatment for Phyllopsora chodatinica

XML Treatment for Phyllopsora cinchonarum

XML Treatment for Phyllopsora confusa

XML Treatment for Phyllopsora cuyabensis

XML Treatment for Phyllopsora dolichospora

XML Treatment for Phyllopsora foliata

XML Treatment for Phyllopsora furfuracea

XML Treatment for Phyllopsora gossypina

XML Treatment for Phyllopsora halei

XML Treatment for Phyllopsora himalayensis

XML Treatment for Phyllopsora isidiosa

XML Treatment for Phyllopsora kalbii

XML Treatment for Phyllopsora loekoesii

XML Treatment for Phyllopsora longiuscula

XML Treatment for Phyllopsora mediocris

XML Treatment for Phyllopsora neofoliata

XML Treatment for Phyllopsora parvifolia

XML Treatment for Phyllopsora parvifoliella

XML Treatment for Phyllopsora porphyromelaena

XML Treatment for Phyllopsora pseudocorallina

XML Treatment for Phyllopsora sabahana

XML Treatment for Phyllopsora santensis

XML Treatment for Phyllopsora siamensis

XML Treatment for Phyllopsora subhispidula

## Footnotes

unpublished data

## Supplementary materials

Supplementary material 1 Uncollapsed version of the tree presented in Fig. 1 Data type: phylogenetic data Explanation note: Extended majority-rule consensus tree resulting from the MrBayes analysis of the mtSSU and ITS alignment with Bayesian PP ≥ 0.7 and/or IQ-TREE maximum likelihood BS ≥ 50 and branch lengths. Strongly supported branches (PP ≥ 0.95 and BS ≥ 75) are marked in bold; branches with PP ≥ 0.95 and BS < 75 or PP < 0.95 and BS ≥ 75 are marked in bold grey; branches only supported with PP ≥ 0.7 or BS ≥ 50 are marked with a dot above the branch. Two species of *Biatora* were used for rooting. Terminal names include DNA extraction number, species name and when relevant, chemotype (ch). Three clades are distinguished to facilitate the discussion of new species (A, B, C). This dataset is made available under the Open Database License (http://opendatacommons.org/licenses/odbl/1.0/). The Open Database License (ODbL) is a license agreement intended to allow users to freely share, modify, and use this Dataset while maintaining this same freedom for others, provided that the original source and author(s) are credited.Sonja Kistenich, Mika Bendiksby, Charles S. Vairappan, Gothamie Weerakoon, Siril Wijesundara, Patricia A. Wolseley, Einar Timdal

Supplementary material 2 Specimens of *Phyllopsora* examined in this study Data type: specimens data This dataset is made available under the Open Database License (http://opendatacommons.org/licenses/odbl/1.0/). The Open Database License (ODbL) is a license agreement intended to allow users to freely share, modify, and use this Dataset while maintaining this same freedom for others, provided that the original source and author(s) are credited.Sonja Kistenich, Mika Bendiksby, Charles S. Vairappan, Gothamie Weerakoon, Siril Wijesundara, Patricia A. Wolseley, Einar Timdal
