# Supplementary figures and images for: A regional study of the genus Phyllopsora (Ramalinaceae) in Asia and Melanesia
Source: MycoKeys. 2019 May 29;53:23–72. doi: 10.3897/mycokeys.53.33425 (PMC6551344; doi:10.3897/mycokeys.53.33425)

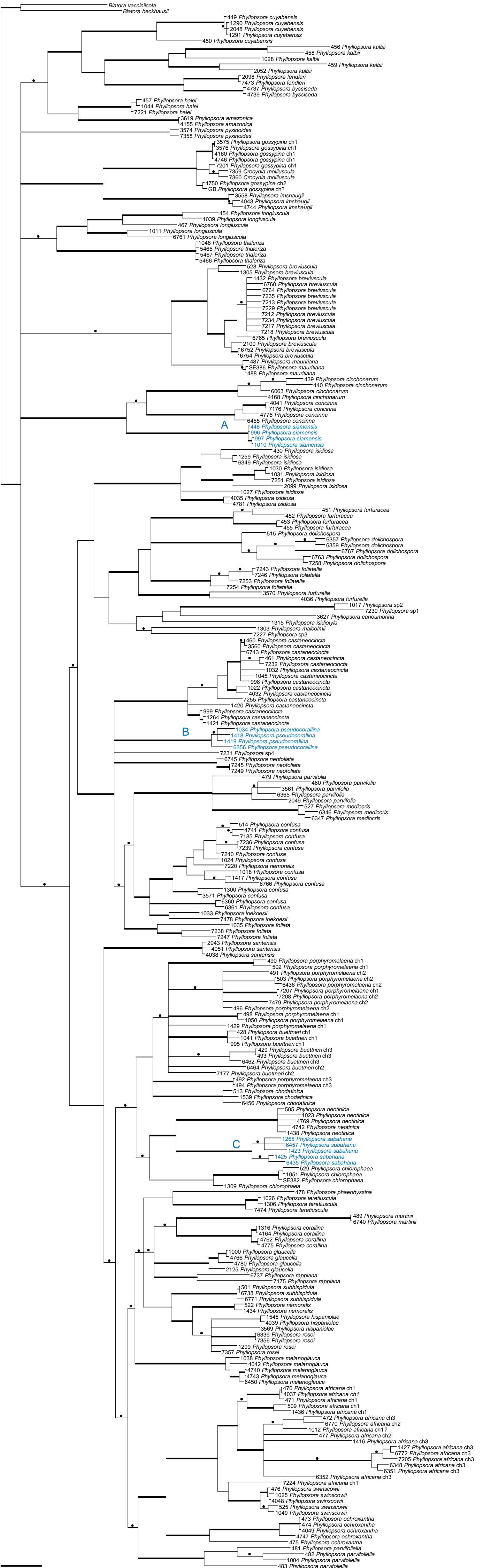

Supplement: Supplementary material 1 [file mycokeys-53-023-s002.pdf]
